# A cuproptosis-related lncRNAs signature for prognosis, chemotherapy, and immune checkpoint blockade therapy of low-grade glioma

**DOI:** 10.3389/fmolb.2022.966843

**Published:** 2022-08-17

**Authors:** Xiuwei Yan, Nan Wang, Jiawei Dong, Fang Wang, Jiheng Zhang, Xueyan Hu, Hongtao Zhao, Xin Gao, Zhihui Liu, Yongzhe Li, Shaoshan Hu

**Affiliations:** ^1^ Department of Neurosurgery, Cancer Center, Zhejiang Provincial People’s Hospital, Affiliated People’s Hospital, Hangzhou Medical College, Hangzhou, China; ^2^ Department of Neurosurgery, The Second Affiliated Hospital of Harbin Medical University, Harbin, China

**Keywords:** cuproptosis, lncRNAs, prognosis, low-grade glioma, immune checkpoint blockade, chemotherapy

## Abstract

Cuproptosis is a new type of cell death that is associated with mitochondrial respiration of the tricarboxylic acid cycle. Previous studies showed that long non-coding RNAs (lncRNAs) regulated low-grade glioma (LGG) progression. However, the potential applications of cuproptosis-related lncRNAs (CRLs) in LGG were not explored. A comprehensive analysis was performed in The Cancer Genome Atlas (TCGA) and Chinese Glioma Genome Atlas (CGGA) cohorts. We first screened two distinct cuproptosis subtypes based on prognostic CRLs using consensus clustering. To facilitate individualized survival prediction in LGG, we constructed a prognostic signature (including CRNDE, HAR1A, and FAM181A-AS1) in the TCGA dataset. The prognostic signature exhibited excellent predictive ability and reliability, which was validated in the CGGA_325 and CGGA_693 datasets. Notably, patients in the high-risk group had increased immune cell infiltration and expression of immune checkpoints, which indicated that they may benefit more from immune checkpoint blockade (ICB) therapy. Finally, the prognostic signature screened the population with sensitivity to chemotherapy and ICB therapy. In summary, this study initially explored the mechanism of CRLs in LGG and provides some insights into chemotherapy and ICB therapy of LGG.

## Introduction

Gliomas are the most common primary intracranial tumor in the central nervous system and are derived from the neuroglial stem or progenitor cells (Weller et al., 2015). Low-grade glioma (LGG, WHO grades II-III) accounts for 13%–16% of gliomas (Chen et al., 2017). Most patients with LGG inevitably progress to high-grade glioma (HGG) (Duffau, 2018). Various treatment methods, such as surgery, radiotherapy, chemotherapy, immunotherapy, electric field therapy, and neutron therapy are used, but tumor recurrence appears to be unavoidable (Lu et al., 2021; Li et al., 2022). Therefore, it is urgent to identify novel potential therapeutic targets for LGG.

Copper is an essential metallic element in the human body, and it is linked to a series of biological processes, such as energy metabolism, autophagy, and oxidative stress (Ge et al., 2022). Since its pivotal role in the genesis, severity, and progression of cancer was recognized, copper has attracted more attention in targeted therapy (Denoyer et al., 2018). A previous study found that copper commonly exhibited anti-tumor effects *via* the formation of metal chelators and ionophores (Steinbrueck et al., 2020). For the first time, Tsvetkov et al. defined Cu-dependent cytotoxicity, which leads to regulated cell death (RCD), as “cuproptosis” (Tsvetkov et al., 2019; Tsvetkov et al., 2022). Previous research suggested that copper-dependent cell death was correlated with the progression and treatment of glioma (Buccarelli et al., 2021). Therefore, this new form of cell death raises hope for successful future therapies for glioma patients.

Long noncoding RNAs (lncRNAs) are a group of transcripts longer than 200 nucleotides that lack protein-coding capacity (Gibb et al., 2011). LncRNAs act as master regulators of gene expression and play vital roles in various biological processes (Peng et al., 2017). LncRNAs are implicated in the progression of stemness, proliferation, angiogenesis, and drug resistance of gliomas (Peng et al., 2018). Up to now, the underlying mechanisms of cuproptosis-related lncRNAs (CRLs) in LGG have not been established.

The present study used genome sequencing technology and bioinformatics analysis to explore the function of CRLs in LGG patients. According to the prognostic CRLs screened from The Cancer Genome Atlas (TCGA) and Chinese Glioma Genome Atlas (CGGA) cohorts, LGG patients were stratified into two cuproptosis subtypes with distinct over survival, clinicopathological features, immune cell infiltration, and biological processes. To quantify the difference between individuals, we established a new prognostic signature based on three CRLs (including CRNDE, HAR1A, and FAM181A-AS1). This prognostic signature had a powerful ability to predict the prognosis of LGG patients. We initially demonstrated the potential of our prognostic signature in predicting immune checkpoint blockade (ICB) therapy and chemotherapy sensitivity.

## Materials and methods

### Data collection and pre-processing

The transcriptional data and clinical information of LGG patients were sourced from the TCGA (https://portal.gdc.cancer.gov) and CGGA (http://www.cgga.org.cn/) datasets. The gene expression profiles of 105 normal brain tissues from the Genome Tissue Expression (GTEx) project was obtained from the UCSC Xena website (https://xena.ucsc.edu/). LGG samples were excluded when they had missing survival information and definitive histopathological diagnosis. Finally, a total of 509 LGG samples from the TCGA dataset and 591 samples from the CGGA dataset (172 from the CGGA_325 cohort and 419 from the CGGA_693 cohort) were filtered for further study. The transcriptional data from the TCGA and CGGA cohorts were TPM normalized. The characteristics of the LGG patients are listed in Supplementary Table S1.

### Identification of the CRLs in the TCGA and CGGA_325 datasets

Seventeen cuproptosis-related genes (CRGs, Supplementary Table S2) were retrieved based on a published study (Tsvetkov et al., 2022). To screen the CRLs, Pearson correlation analysis was performed between the CRGs and lncRNAs in the TCGA and CGGA_325 cohorts. An absolute value of correlation coefficients >0.5 corresponding to a *p*-value < 0.05 was considered eligible.

### Consistent clustering to determine the cuproptosis subtypes

Univariate Cox regression analysis was used to screen the prognostic CRLs. The prognostic CRLs shared by the TCGA and CGGA_325 cohorts were selected for unsupervised clustering according to the R package “ConsensusClusterPlus”. The candidate cluster number ranged from two to six. To produce the most stable consensus matrix, the procedure was repeated 100 times with an 80% sample selected in each iteration (Wilkerson and Hayes, 2010).

### Evaluation of the immune landscape

Infiltrating immune and stromal cells are mainly structural components of the tumor microenvironment (TME). We used the Estimation of STromal and Immune cells in MAlignant Tumor tissues using Expression data (ESTIMATE) algorithm to calculate the abundance of immune and stromal cells in each LGG patient (Yoshihara et al., 2013).

To better understand immune cell infiltration in the TME of LGG samples, the Tumor Immune Estimation Resource (TIMER) algorithm (Li et al., 2017) was used in the “IOBR” R package (Zeng et al., 2021). For validation, the single-sample gene set enrichment analysis (ssGSEA) algorithm was also used to evaluate the relative abundance of infiltrating immune cells. Jia et al. (2018)summarized the characteristic gene panels for TME-infiltrating immune cell types (Supplementary Table S3).

Tumor Immunophenotype Profiling (TIP, http://biocc.hrbmu.edu.cn/TIP/index.jsp) is a web-based tool that makes the process of anticancer immunity easy to visualize (Xu et al., 2018). According to the TIP, the anti-tumor immune response may be simplified as a seven-step cycle event (Supplementary Table S4): the release of cancer cell antigens (Step 1), cancer antigen presentation (Step 2), priming and activation (Step 3), trafficking of immune cells to tumors (Step 4), infiltration of immune cells into tumors (Step 5), recognition of cancer cells by T cells (Step 6), and killing of cancer cells (Step 7). In this study, we assessed the anti-tumor activity score of each LGG sample in the TCGA database.

### Functional enrichment analyses

Gene set variation analysis (GSVA) is a functional enrichment analysis that estimates the difference in pathway activity of the samples using an unsupervised method (Hänzelmann et al., 2013). To investigate the difference in biological processes between distinct clusters, we performed GSVA using the “GSVA” package in R. Kyoto Encyclopedia of Genes and Genomes (KEGG) gene sets were obtained from the Molecular Signatures Database (MSigDB, http://www.gsea-msigdb.org/gsea/index.jsp, v7.5.1) (Liberzon et al., 2011). KEGG pathways between different subtypes with a false discovery rate (FDR) < 0.05 were considered significant. Sixteen gene sets that represent classical biological processes were also analyzed for validation (Supplementary Table S5) (Mariathasan et al., 2018).

### Chemotherapeutic and immune checkpoint blockade therapy response prediction

LGG patients greatly benefit from chemotherapy (Shaw et al., 2012; Viaccoz et al., 2012). According to the Genomics of Drug Sensitivity in Cancer (GDSC, https://www.cancerrxgene.org/) database, the “pRRophetic” package in R was used to evaluate the chemotherapy drug sensitivity of each LGG sample (Geeleher et al., 2014). Four commonly used chemotherapeutics (temozolomide, tamoxifen, bleomycin, and vinblastine) were selected in the present study.

The subclass mapping method from GenePattern (SubMap, https://www.genepattern.org/) was used to predict the response to ICB therapy (Hoshida et al., 2007). The SubMap method is an unsupervised algorithm that reveals common subtypes between independent datasets. The gene expression profiles of 47 melanoma patients who received ICB therapy were sourced from a published dataset (Roh et al., 2017).

### Identification of differentially expressed cuproptosis-related lncRNAs between cuproptosis subtypes

According to the “limma” package in R (Ritchie et al., 2015), we further analyzed the differential expression of 37 prognostic CRLs between distinct cuproptosis subgroups. Differentially expressed CRLs (DE-CRLs) with an FDR <0.05 and | log2fold change (FC) | values >1 were considered significant.

### Construction and validation of cuproptosis-related lncRNAs signature

The overlapping DE-CRLs from the TCGA and CGGA_325 cohorts were incorporated into the least absolute shrinkage and selection operator (LASSO) regression analysis using the “glmnet” R package (Friedman et al., 2010). The risk score for each sample was calculated by the expression level and regression coefficient of each CRL. The formula is described below:

$$Risk\;score\;=\overset{n}{\underset{i=1}{\sum }}Coef\left(X_{i}\right)*\;Exp\;\left(X_{i}\right)$$

Coef *(X*_*i*_ *)* is the regression coefficient of the CRLs, and Exp *(X*_*i*_ *)* represents the expression levels of CRLs. LGG samples were split into high- and low-risk score groups by the median value.

The Kaplan-Meier (K-M) survival curve was employed to compare the overall survival between the high- and low-risk groups. The receiver operating characteristic (ROC) curves were drawn to measure the predictive power of cuproptosis-related lncRNAs signature through the “survival-ROC” R package (Heagerty et al., 2000).

### Nomogram construction

Univariate and multivariate Cox regression analyses were used to evaluate the independent prognostic value of cuproptosis-related lncRNA signature. To evaluate the Cox regression model, the PH hypothesis test was performed. Nomogram was constructed to predict the survival probability of 1-, 3-, and 5-years overall survival (Iasonos et al., 2008). The calibration plot was also performed to verify the accuracy.

### Real-time quantitative PCR

Four tumor tissues and corresponding peritumoral brain tissues from LGG patients were collected in the Second Affiliated Hospital of Harbin Medical Universit. This research was approved by all the patients and the Ethics Committee of the hospital. Total RNA was isolated from LGG tissues using TRIzol reagent (Invitrogen, United States) according to the manufacturer’s protocol. According to the manufacturer’s instructions of the Nanodrop ND-2000 spectrophotometer (Thermo Scientific, United States), 2 μg of the total RNA was transcribed into cDNA. RT-qPCR was performed with the SYBR Green PCR kit (Takara, Japan). Independent experiments were conducted in triplicate, and ACTB served as an internal control. The primers (Tsingke Biotechnology Co., Ltd, Beijing, China) were used are displayed in Supplementary Table S6.

### Statistical analysis

All statistical analyses and visualizations were executed in R 4.1.2 and GraphPad Prism 8.0.2. Survival analysis was performed using the “survival” package in R. The nomogram and the calibration curves were generated by the “rms” R package (Zhang and Kattan, 2017). The R package “maftools” was used to process and present the mutation data (Mayakonda et al., 2018). Immune cell infiltration, ssGSEA score, mRNA expression, and TIP score were compared between the two groups using the Wilcoxon test. The student’s *t*-test was used to analyze differences between different risk groups. *P* < 0.05 was considered statistically significant.

## Results

### Identification of prognostic CRLs from TCGA and CGGA_325 datasets

**FIGURE 1:**
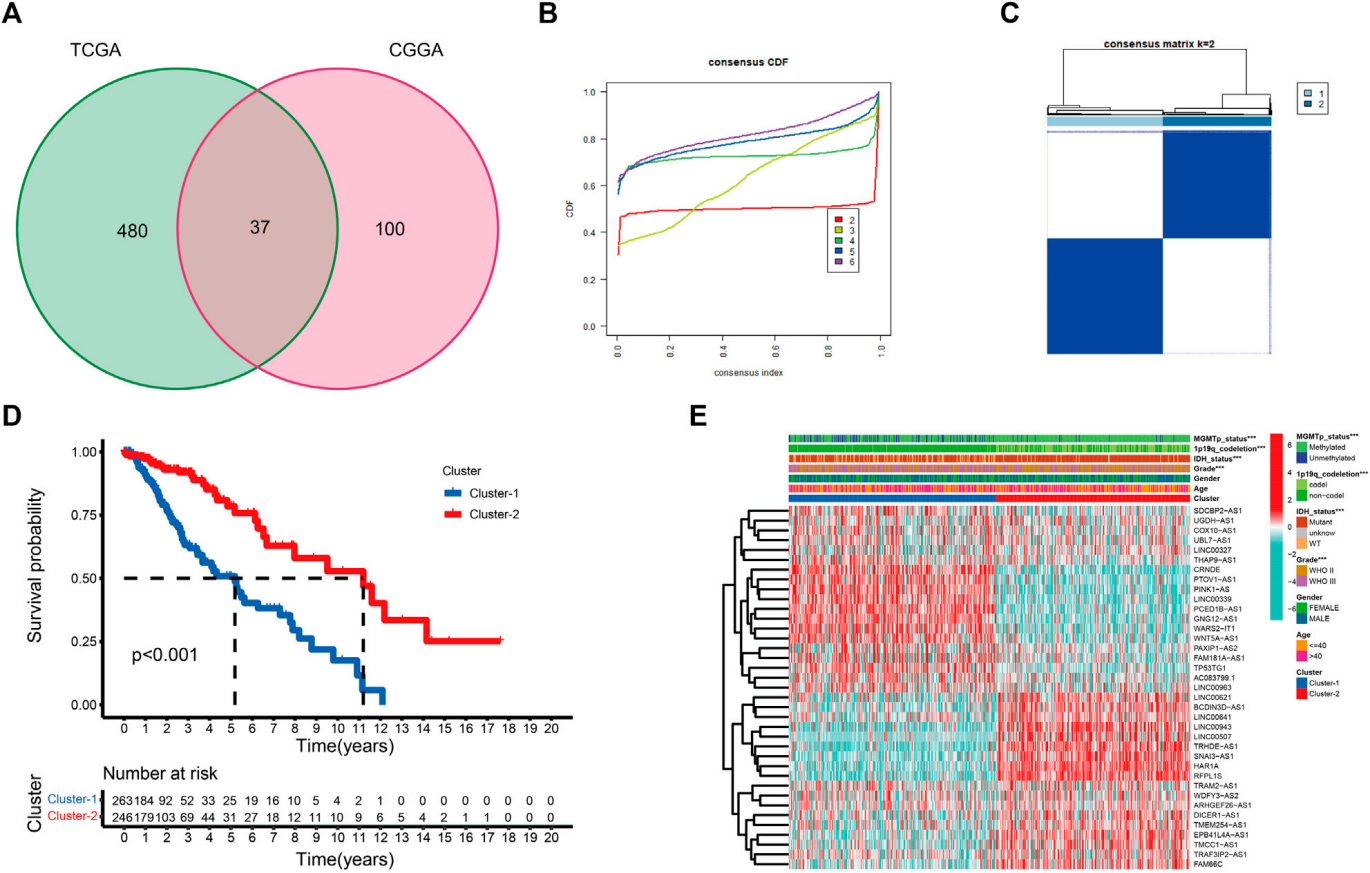
**(A)** Venn diagram to identify the intersecting prognostic CRLs from TCGA and CGGA_325 cohorts. **(B)** Cumulative distribution function curves for *k* = 2–6. **(C)** Consensus clustering matrix for *k* = 2. **(D)** K-M curve between two cuproptosis subtypes. **(E)** The expression profile of 37 CRLs between cuproptosis subtypes in the TCGA cohort (*p < 0.05, **p < 0.01, and ***p < 0.001).

Supplementary Figure S1 provides a flow chart of this study. Pearson correlation analysis was performed between CRGs and CRLs. Combined with univariate Cox regression analyses, a total of 517 and 137 CRLs were obtained from the TCGA and CGGA_325 datasets, respectively, using | cor | > 0.5 and *p* < 0.05 as cutoff values (Supplementary Tables S7, S8). Thirty-seven prognostic CRLs that were shared by the two datasets were screened (Figure 1A).

### Determine the cuproptosis subtypes in LGG patients

To better examine the role of cuproptosis in LGG patients, unsupervised consensus clustering of the 37 prognostic CRLs was performed using the R package “Consensus ClusterPlus”. As shown in the cumulative distribution function (CDF) plots and consensus matrix, *k* = 2 was the most suitable choice (Figures 1B,C). We obtained two cuproptosis subtypes from the TCGA dataset, which were labeled cluster-1 and cluster-2. A total of 263 samples were classified into cluster-1, and 246 samples were classified into cluster-2 (Supplementary Table S9). To confirm the robustness of unsupervised clustering, the same algorithm was used in the CGGA_325 cohort (Supplementary Figures S2A,B). We also obtained two distinct subtypes. Eighty samples were classified into cluster-1, and 92 samples were classified into cluster-2 (Supplementary Table S10). These results demonstrated the effectiveness of our grouping.

K-M survival curves showed that patients in cluster-2 had a significant survival advantage (Figure 1D; Supplementary Figure S2C). The distribution of various clinical factors and 37 CRL expression levels between different subgroups are intuitively shown in Figure 1E. Compared to cluster-2, patients in cluster-1 were more related to the clinicopathological features of WHO III, isocitrate dehydrogenase (IDH) wild-type, unmethylated O6-methylguanine-DNA methyltransferase promoter (MGMTp), and 1p19q non-codeletion. A similar result was observed in the CGGA_325 cohort (Supplementary Figure S2D).

### Tumor microenvironment immune cell infiltration in different cuproptosis subtypes

**FIGURE 2:**
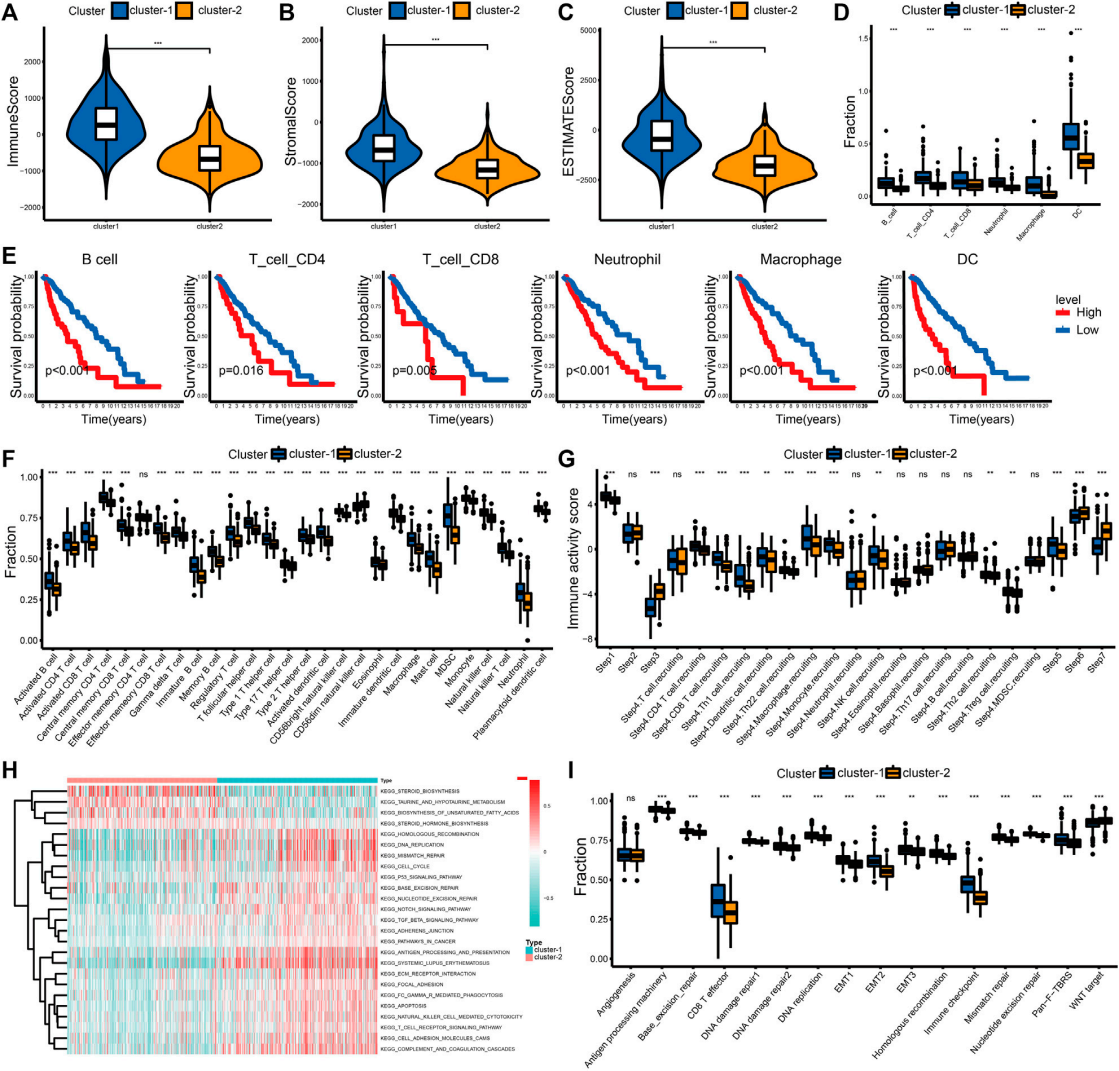
Differential immune landscape and biological processes between cuproptosis subtypes in the TCGA cohort. **(A–C)** Distribution of immune score **(A)**, stromal score **(B)**, and ESTIMATE score **(C)** between the two cuproptosis subtypes. **(D)** Estimated abundance of six immune cells from the TIMER algorithm. **(E)** The K-M curves for patients in the high- and low-level immune cell groups from the TIMER algorithm. **(F)** Estimated abundance of 28 immune cells from the ssGSEA algorithm. **(G)** Immune activity scores of the anti-tumor immune response. **(H)** Heatmap of KEGG pathways between cuproptosis subtypes. **(I)** Comparison of 16 classical biological processes between the cuproptosis subtypes. The horizontal line of the box plot represents the median values (*p < 0.05, **p < 0.01, ***p < 0.001 and ns, non-significant).

We examined the difference in immune cell infiltration between different cuproptosis subgroups. The immune and stromal scores positively correlated with the number of immune or stromal components in the TME. The ESTIMATE score represents the comprehensive proportion of the immune and stromal scores in the TME (Yoshihara et al., 2013). The immune score, stromal score, and ESTIMATE score were significantly higher in cluster-1 in this study, which indicates an increased infiltration of immune and stromal cells (Figures 2A–C; Supplementary Figures S3A–C). Based on the TIMER algorithm, we calculated the abundance of six immune cells, including B cells, CD4^+^ T cells, CD8^+^ T cells, neutrophils, macrophages, and myeloid dendritic cells, in the TME. The infiltration levels of six immune cells in cluster-1 were significantly increased (Figure 2D; Supplementary Figure S3D). Patients with higher immune cell infiltration corresponded with poor outcomes in LGG (Figure 2E; Supplementary Figure S3E). We also evaluated the infiltration levels of 28 immune cells using the ssGSEA score to validate these results (Figure 2F; Supplementary Figure S3F).

To better understand the anti-tumor immune response in LGG, the process was visualized using the TIP website. Patients in cluster-1 had increased immune activity scores in Step 1, Step 4, and Step 5. However, the immune activity scores in Step 3, Step 6, and Step 7 of cluster-2 were higher (Figure 2G). Although the recruitment and infiltration of immune cells in cluster-1 were higher, the abilities to recognize and kill cancer cells were lower than in cluster-2. This difference also explained why the increasing level of immune infiltration was associated with shorter overall survival in LGG.

### Characteristics of the biological process in distinct cuproptosis subtypes

To further examine the differences in biological processes between distinct cuproptosis subtypes, we performed the GSVA enrichment analysis. Cluster-1 was markedly enriched in stromal, immune activation, oncogenic, and DNA damage repair (DDR) pathways, such as the ECM receptor interaction, antigen processing and presentation, TGF-β signaling pathway, and DNA replication. Patients in cluster-2 were enriched in pathways related to metabolism, including taurine and hypotaurine metabolism and the biosynthesis of unsaturated fatty acids (Figure 2H; Supplementary Table S11). Differences in 16 typical biological processes between cluster-1 and cluster-2 were also identified in this study. The stromal, immune activation, and DDR pathways were enriched in cluster-1 (Figure 2I). These results were verified in the CGGA_325 cohort (Supplementary Figures S3G,H, Supplementary Table S12).

### Chemotherapy sensitivity and the immune checkpoint blockade treatment responsiveness in different cuproptosis subtypes

**FIGURE 3:**
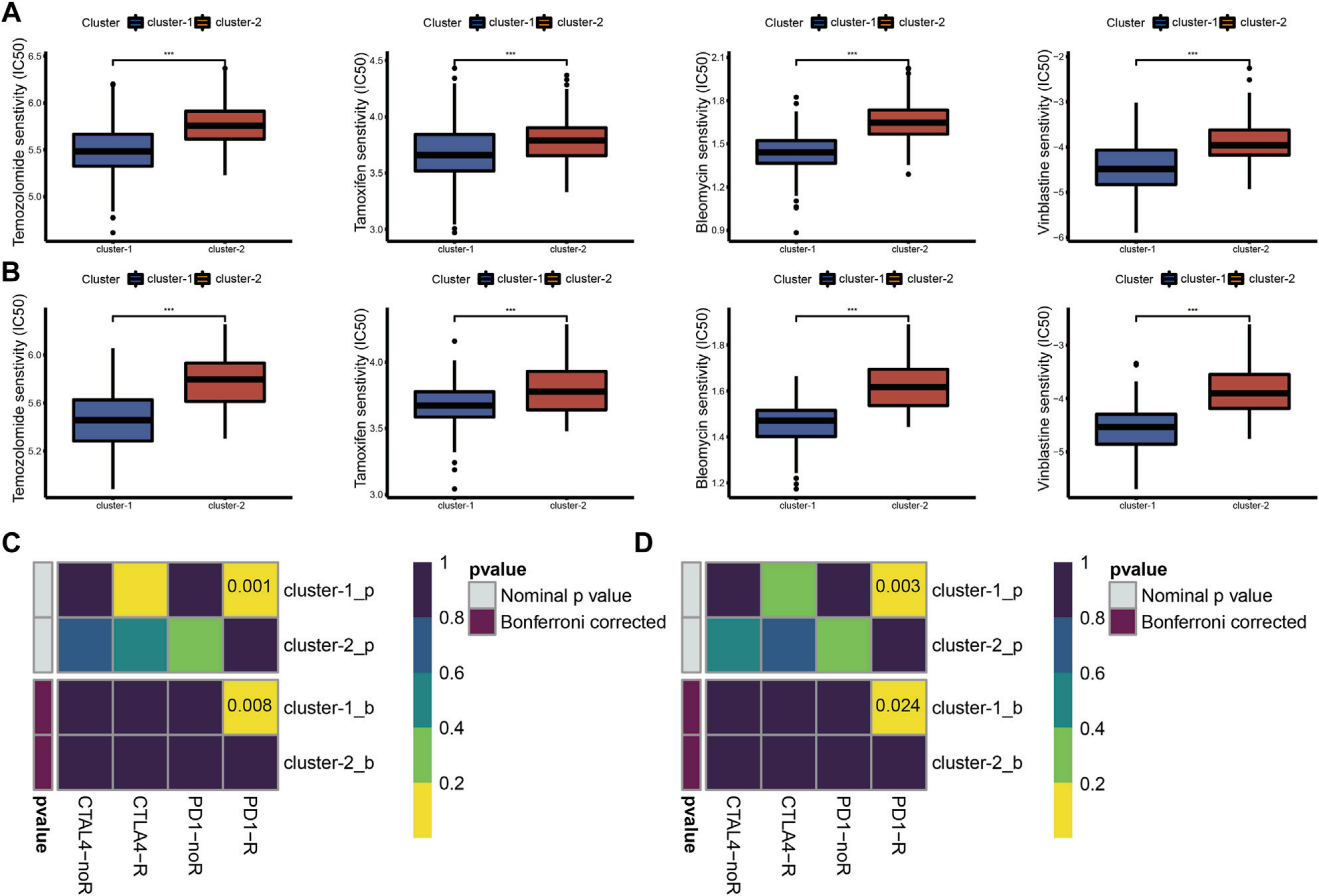
Drug sensitivity of temozolomide, tamoxifen, bleomycin, and vinblastine between cuproptosis subtypes in TCGA **(A)** and CGGA_325 **(B)** cohorts. ICB therapy responses between cuproptosis subtypes in TCGA **(C)** and CGGA_325 **(D)** cohorts (*p < 0.05, **p < 0.01, ***p < 0.001 and ns, non-significant).

Chemotherapy is the main adjuvant therapy for LGG, and it provided great benefit. The present study assessed the half-maximal inhibitory concentration (IC_50_) of four commonly used chemotherapeutics (temozolomide, tamoxifen, bleomycin, and vinblastine) in each LGG sample. Patients in cluster-1 had a higher sensitivity for four chemotherapeutics (Figures 3A,B).

The abundance of infiltrating immune cells and enrichment of immune checkpoint pathways in cluster-1 indicated a positive response to ICB therapy. Considering the importance of ICB therapy in LGG, we next examined the response to ICB therapy in LGG. The results demonstrated that patients in cluster-1 were more responsive to anti-PD-1 treatment (Figures 3C,D).

### Construction of the cuproptosis-related lncRNAs signature

**FIGURE 4:**
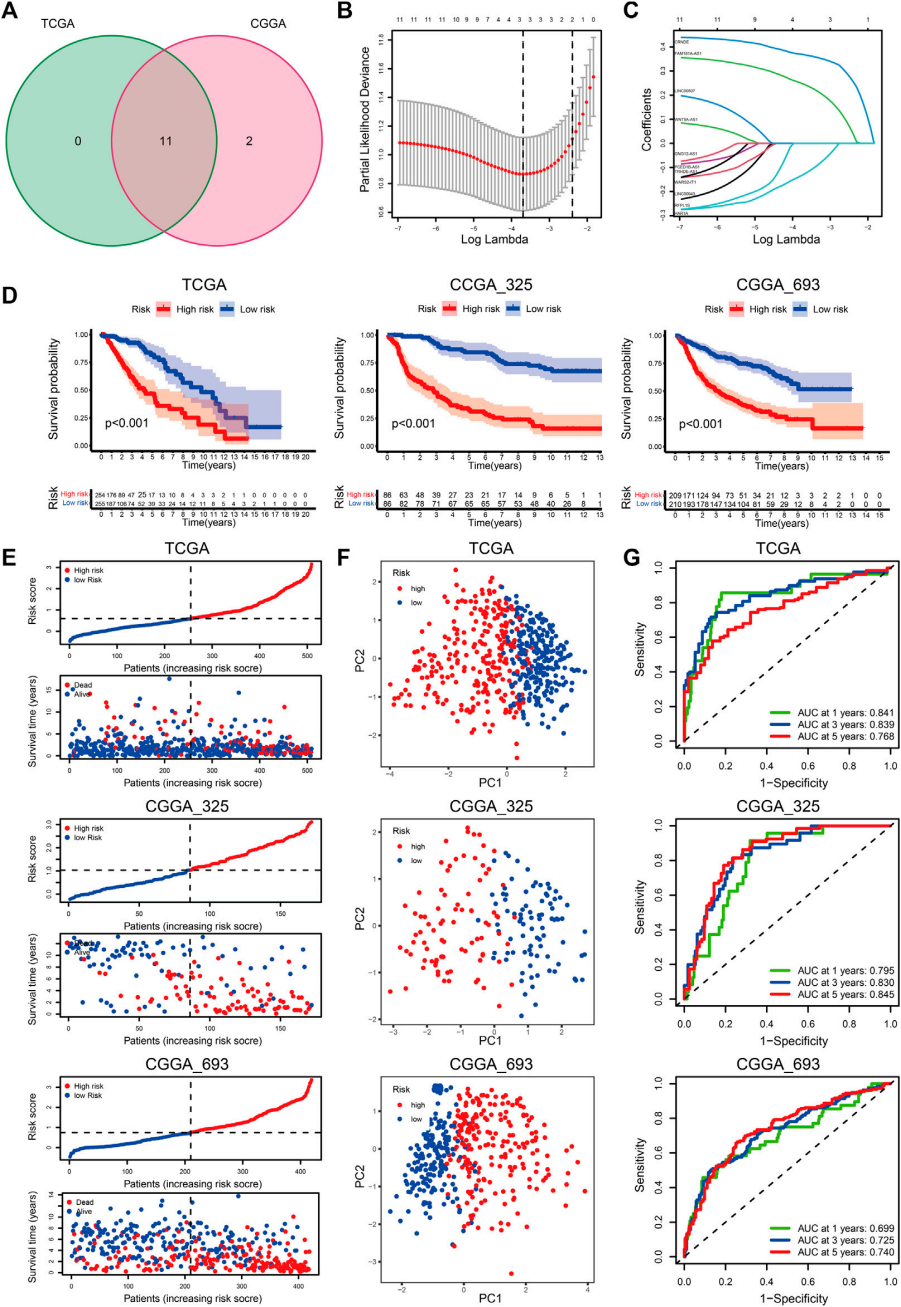
**(A)** Venn diagram to identify the intersecting DE-CRLs from TCGA and CGGA_325 cohorts. **(B)** Ten-time cross-validation for tuning parameter selection in the LASSO model. **(C)** LASSO coefficient profiles of the 3 prognostic CRLs. **(D–G)** The K-M curves between the high- and low-risk groups **(D)**, PCA plots **(E)**, distribution plots of the risk score and survival status **(F)**, and time-dependent ROC analysis **(G)** in the TCGA, CGGA_325, and CGGA_693 datasets.

With an FDR <0.05 and | logFC | > 1, we obtained eleven and thirteen DE-CRLs from the TCGA and CGGA_325 datasets, respectively (Supplementary Table S13). Eleven overlapped prognostic CRLs were selected from the two cohorts (Figure 4A). Considering the individual heterogeneity, we established a CRL-based prognostic model to quantify the difference between the individuals in LGG (Figures 4B,C). The regression coefficients of the three CRLs are shown in Supplementary Table S14. We divided the LGG patients into low- and high-risk score groups at the median cut-off. Patients in cluster-1 showed an increased risk score compared to cluster-2 (Supplementary Figures S4A, S5A). The clinicopathological features of age ≤ 40 years, WHO II, 1p19q codeletion, IDH mutation, and MGMTp methylation were associated with a decreased risk score (Supplementary Figures S4B–E, S5B–E, S6B–E).

K-M curves indicated that patients with a high-risk score had a poor prognosis (Figure 4E). The distribution plot of the risk score and survival status showed that patients in the low-risk group had a higher survival rate (Figure 4F). Principal component analysis (PCA) showed that the LGG patients were easily distinguished according to the different risk groups (Figure 4G). The AUC values of the signature for predicting 1-, 3-, and 5-years survival rates in the TCGA dataset were 0.841, 0.839, and 0.768, respectively. The AUC values were 0.773 (1-year), 0.834 (3-years), and 0.849 (5-years) in the CGGA_325 cohort, and the AUC values in the CGGA_693 cohort were 0.699, 0.725, and 0.740 for 1, 3, and 5 years, respectively. (Figure 4H).

**FIGURE 5:**
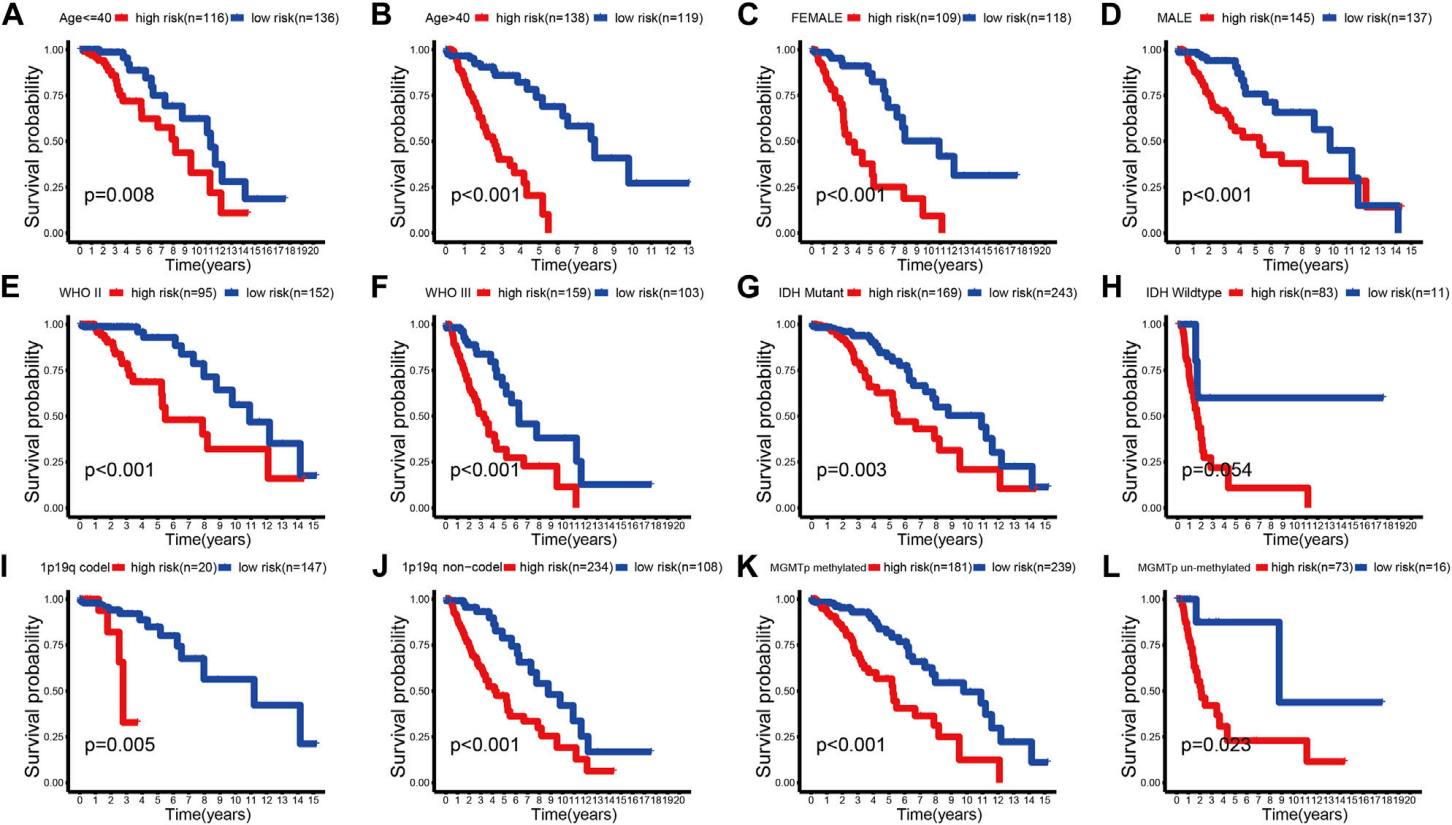
K-M survival curves of the high- and low-risk groups stratified into multiple TCGA clinical subgroups. **(A,B)** Age ≤ 40 or >40 years. **(C,D)** Female or male. **(E,F)** WHO II or WHO III. **(G,H)** IDH mutant or wild type. **(I,J)** 1p19q codel or no-codel. **(K,L)** MGMTp methylate or non-methylate.

We further evaluated the predictive ability of the prognostic signature between different clinical subgroups. The results showed that patients in the low-risk group always had a better outcome than patients in the high-risk group (Figure 5A–L; Supplementary Figures S7A–L, S8A–L). These findings demonstrated the favorable predictive ability of the prognostic signature.

### Building a predictive nomogram for overall survival prediction

**FIGURE 6:**
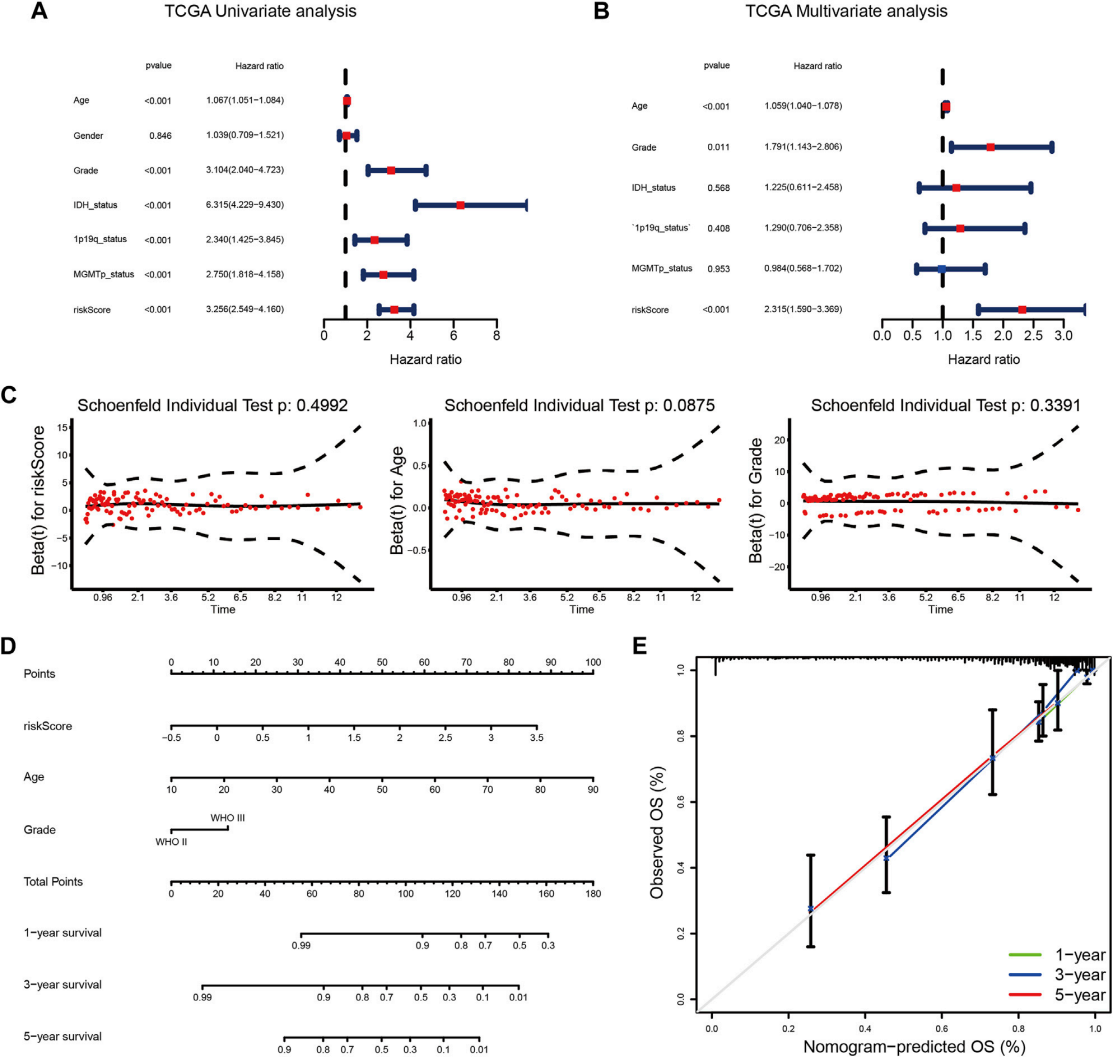
**(A,B)** Univariate **(A)** and multivariate **(B)** Cox analyses of the prognostic signature in the TCGA cohort. **(C)** PH hypothesis test for each independent prognostic factor. **(D)** A nomogram for predicting the 1-, 3-, and 5-years survival rates of LGG patients in the TCGA cohort. **(E)** The calibration curves predicted 1-, 3-, and 5-years survival rates in the TCGA cohort.

The results of univariate and multivariate Cox regression analyses showed that the risk score was always an independent prognostic factor in LGG (Figures 6A,B; Supplementary Figures S9A,B; Supplementary Figures S10A,B). After the PH hypothesis test (Figure 6C; Supplementary Figures S9C, S10C), a nomogram prediction model was constructed (Figure 6D; Supplementary Figures S9D, S10D). We found that the calibration curves of the nomogram were close to the standard curves (Figure 6E; Supplementary Figures S9E, S10E). These results also indicated the clinical applicability of the nomogram.

### Tumor microenvironment immune cell infiltration in different risk groups

**FIGURE 7:**
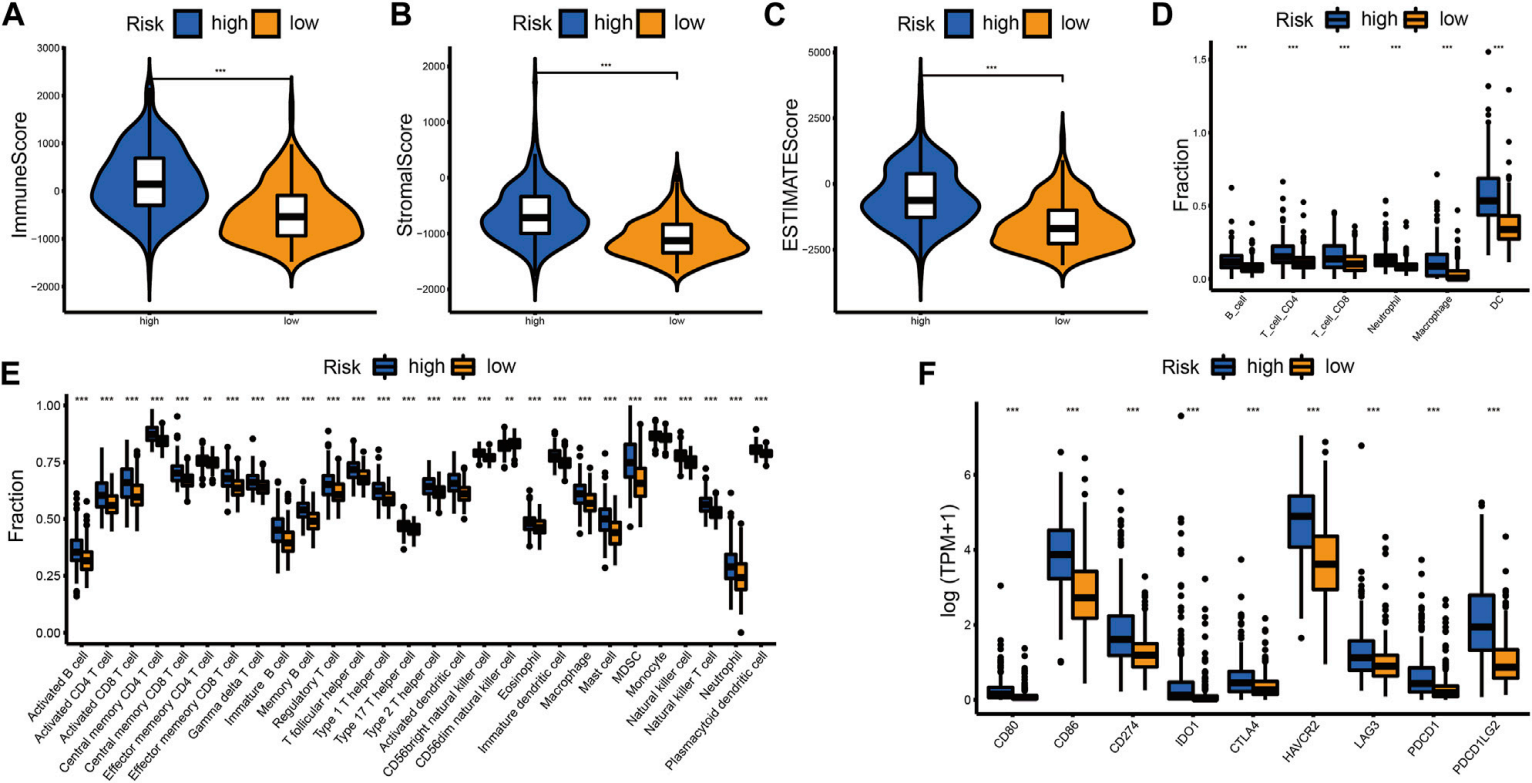
Differential immune landscape between the high- and low-risk groups in the TCGA cohort. **(A–C)** Distribution of immune score **(A)**, stromal score **(B)**, and ESTIMATE score **(C)** between the high- and low-risk groups. **(D)** Estimated abundance of six immune cells from the TIMER algorithm. **(E)** Estimated abundance of 28 immune cells from the ssGSEA algorithm. **(F)** The expression level of ICPs between the high- and low-risk groups. The horizontal line of the box plot represents the median values (*p < 0.05, **p < 0.01, ***p < 0.001 and ns, non-significant).

The difference in immune landscape between different risk groups was also assessed in this research. Patients in the high-risk group had a higher immune score, stromal score, and ESTIMATE score (Figures 7A–C; Supplementary Figures S11A–C, S12A–C). The abundance of immune cell infiltration in the high-risk group was also increased compared to the low-risk group (Figures 7D,E; Supplementary Figures S11D,E, Supplementary Figures S12D,E). We examined the expression levels of immune checkpoint (ICP) regulators including CD80, CD86, CD274 (PD-L1), IDO1, CTLA4, HAVCR2 (TIM-3), LAG3, PDCD1 (PD-1), and PDCD1LG2 (PD-L2) in the different risk groups. We found that most of the ICPs were upregulated in the high-risk group, which indicated that more benefits may be gained from ICB therapy (Figure 7F; Supplementary Figures S11F, S12F).

### Relationship between prognostic signature and mutational status

**FIGURE 8:**
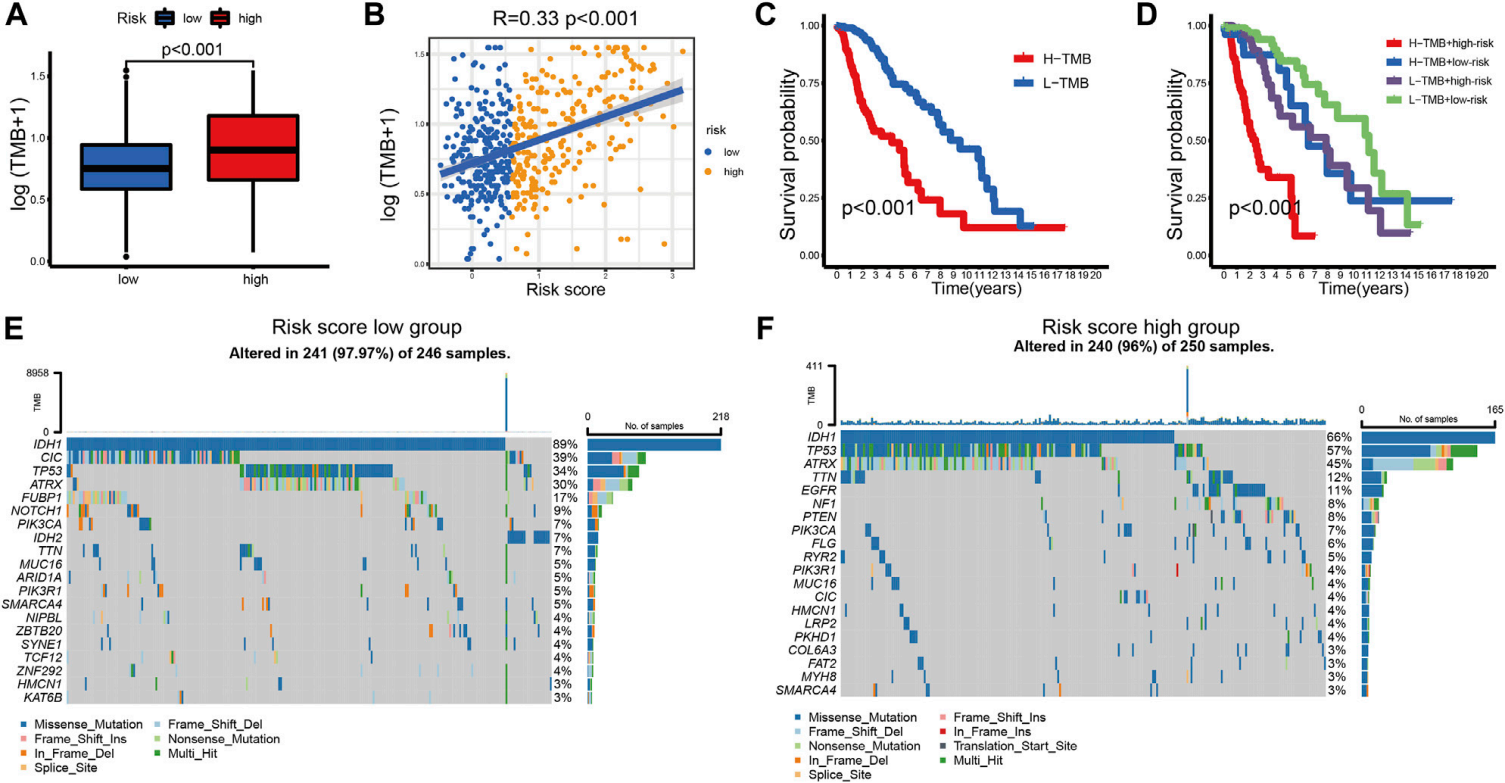
**(A)** TMB levels between the high- and low-risk groups. **(B)** Correlation between TMB levels and risk score in LGG patients. **(C)** The K-M curves of LGG patients in the TMB level-high and -low groups. **(D)** The K-M curves of the four subgroups based on the risk score and TMB levels. **(E,F)** The mutation rates of the top 20 mutated genes of the low- **(E)** and high-risk groups **(F)**.

Tumor mutation burden (TMB) has been used to predict prognosis and ICB efficacy across many cancer types (Snyder et al., 2014; Rizvi et al., 2015; Hugo et al., 2016). To better understand the role of TMB in LGG, we calculated the TMB level of each sample in LGG. Patients in the high-risk group had a higher TMB level (Figure 8A). The TMB levels increased with the risk score (Figure 8B). We also found that the TMB level negatively correlated with overall survival (Figure 8C). Patients in the low-risk group showed an apparent survival advantage in the high and low TMB groups (Figure 8D).

The top 20 mutated genes in different risk groups were visualized using waterfall plots (Figures 8E,F). IDH mutation is an important factor that is associated with favorable outcomes in LGG patients (Choi et al., 2021). Glioblastoma with wild-type IDH may directly transform from LGG with wild-type IDH (Brat et al., 2015). Among the top 20 mutated genes, patients in the low-risk group (IDH1-89%, IDH2-7%) had a higher IDH mutation rate than patients in the high-risk group (IDH1-66%). This result supports the central role of IDH mutation in LGG. CIC mutation was also highly expressed in the low-risk group, which is associated with better survival of glioma (Hwang et al., 2020). The mutation rates of potential targets, including TP53, ATRX, EGFR, and TTN, were higher in the high-risk group (Chen et al., 2016; Jia et al., 2019), which indicated a positive response to ICB therapy.

### Chemotherapy sensitivity and the immune checkpoint blockade treatment responsiveness in different risk groups

**FIGURE 9:**
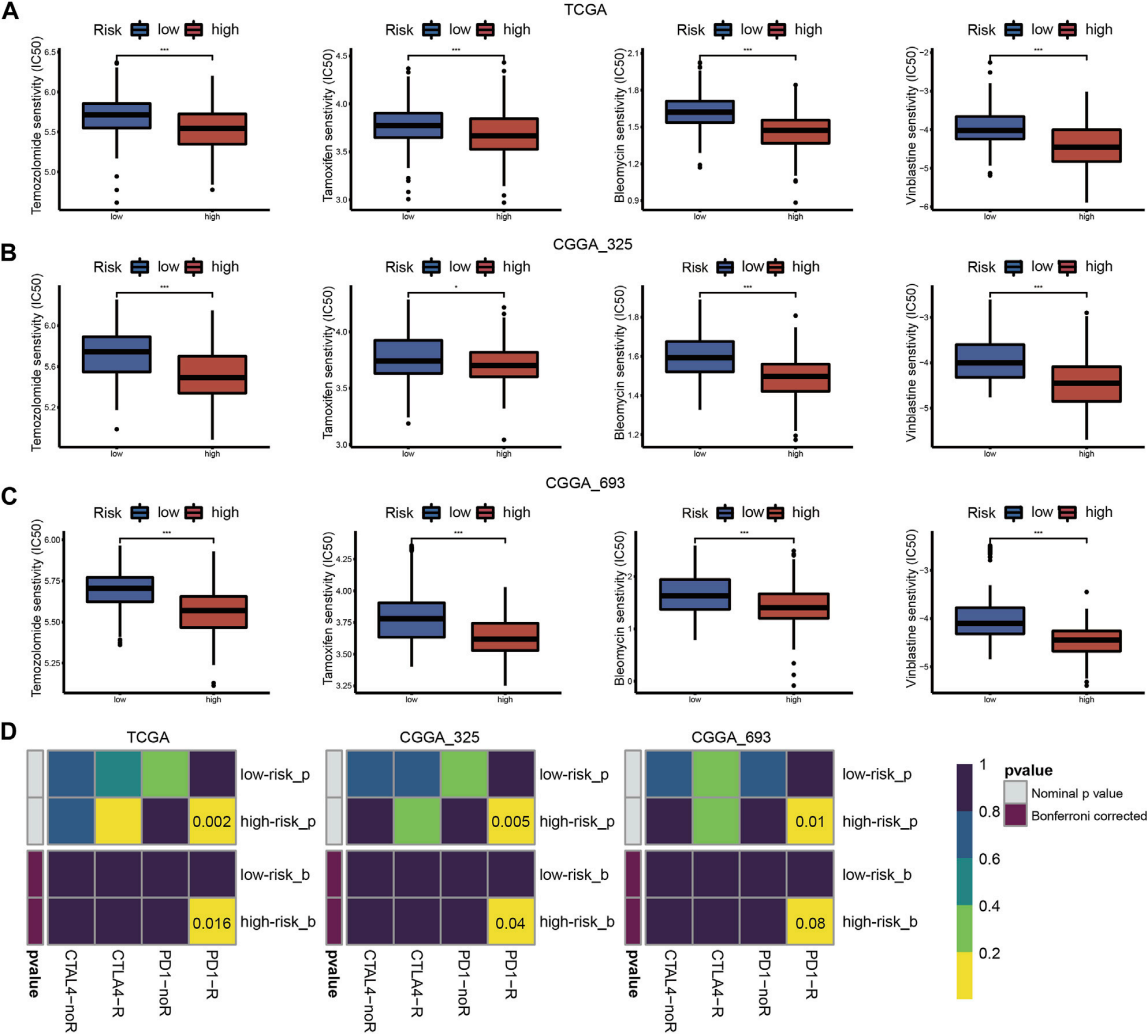
Drug sensitivity of temozolomide, tamoxifen, bleomycin, and vinblastine between high- and low-risk groups in the TCGA **(A)**, CGGA_325 **(B)** and CGGA_693 **(C)** cohorts. **(D)** ICB therapy responses between high- and low-risk groups in the TCGA, CGGA_325 and CGGA_693 cohorts (*p < 0.05, **p < 0.01, ***p < 0.001 and ns, non-significant).

We assessed the predictive value of the risk score for chemotherapy and ICB therapy to help construct individualized treatment plans. The IC50 values of the four chemotherapy drugs showed that patients in the high-risk group were more sensitive to chemotherapy (Figures 9A–C). As shown in Figure 9D, patients in the high-risk group may have greater benefits from ICB therapy.

### Validation of the 3 CRLs expressions in LGG tissue samples

**FIGURE 10:**
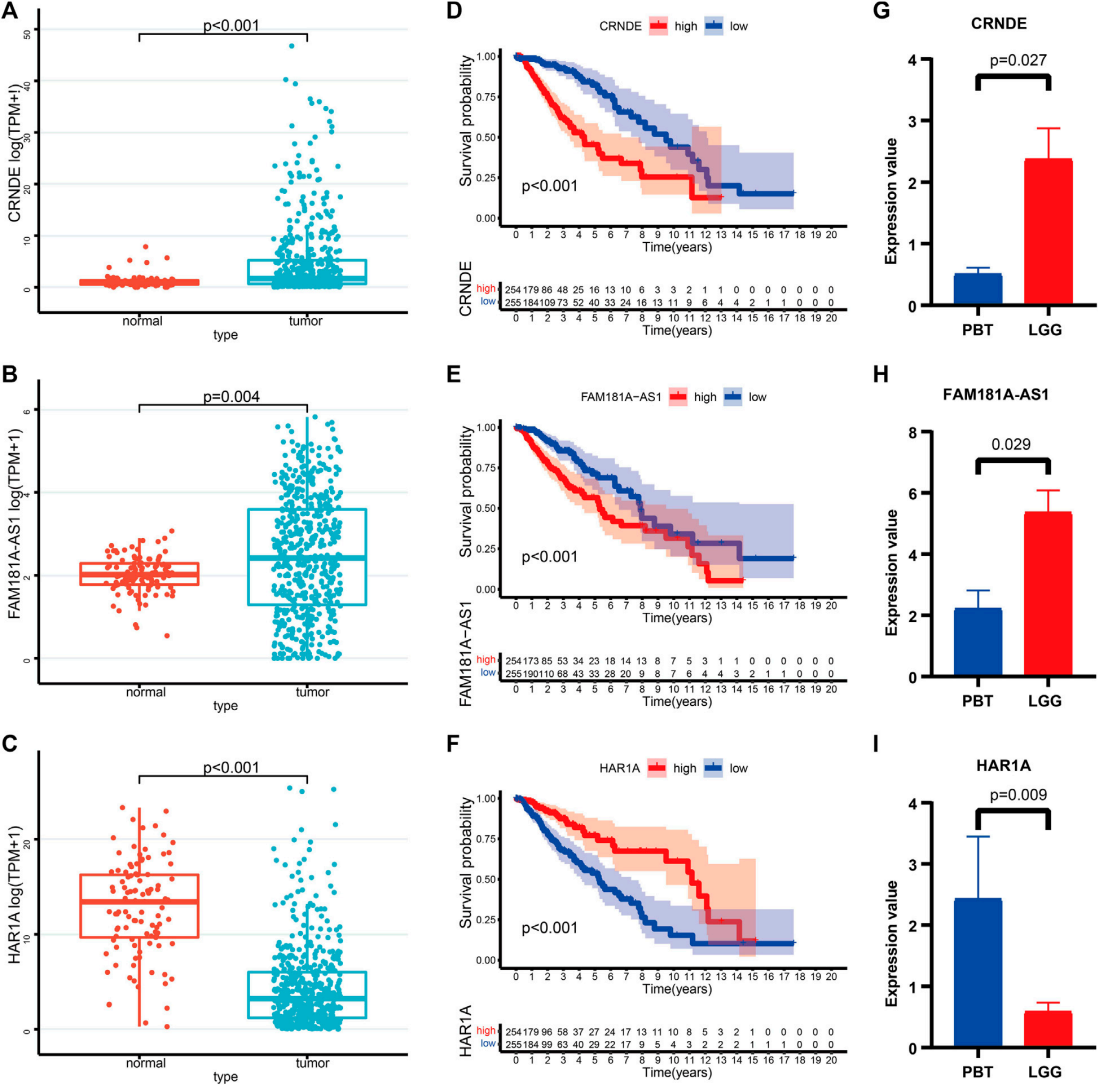
**(A–C)** The expression levels of CRNDE **(A)**, FAM181A-AS1 **(B)**, and HAR1A **(C)** between normal brain tissues and LGG. **(D–F)** K-M survival curves of the high- and low levels of CRNDE **(D)**, FAM181A-AS1 **(E)**, and HAR1A **(F)** in LGG patients. **(G–I)** Relative expression of CRNDE **(G)**, FAM181A-AS1 **(H)**, and HAR1A **(I)** was detected by qRT-PCR in four pairs of LGG and PBT.

We further characterized these 3 CRLs based on gene expression and survival prognosis using the TCGA and GTEx data. As is shown in Figures 10A–C, CRNDE, and FAM181A-AS1 were expressed at high levels in LGG tissues, while HAR1A was increased in normal brain tissues. K-M curves proved the clinical outcomes between different expression levels of 3 CRLs (Figures 10D–F). We further verified the expression levels of 3 CRLs between LGG and peritumoral brain tissues (PBT) with RT-qPCR (Figures 10G–I).

## Discussion

Compared to the most malignant glioblastoma, the median survival of LGG is longer. However, LGG generally converts into high-grade glioma approximately 4–5 years after diagnosis despite receiving standard care (Kumthekar et al., 2015). Cuproptosis is a form of copper-dependent cell death that is different from known cell death pathways. Dysregulated copper metabolism has been found in many types of cancer, which suggests an irreplaceable characteristic in the development of cancer (Kucharzewski et al., 2003; Jouybari et al., 2020). LncRNAs participate important roles in tumor incidence, development, and metastasis (Tang et al., 2022). The underlying mechanism of CRLs in LGG is not clear. To the best of our knowledge, this report is the first study to assess the correlation between CRLs and biological and clinical features of LGG using a bioinformatics method.

The present study first screened 37 prognostic CRLs from the TCGA and CGGA cohorts using the CRGs. According to the consistent clustering, samples in the two datasets were divided into two subtypes with different expression levels of CRLs. Patients in different cuproptosis subgroups had distinct prognostic and clinicopathological features. Cluster-1 was enriched in the stromal, immune activation, oncogenic, and DDR pathways. In contrast, patients in cluster-2 were more active in pathways related to metabolism. Therefore, combined with a previous study on cuproptosis in glioma, patients in cluster-1 were regarded as the cuproptosis-resistant type. Cluster-2 patients were characterized as a cuproptosis-sensitive type.

We also found that patients in cluster-1 had increased immune cell infiltration. The abundance of immune cells was negative for the overall survival of LGG. One of the most important functions of dendritic cells (DCs) is antigen uptake and processing. Using the antigen-processing machinery (APM), tumor-derived epitopes are cross-presented to T cells by DCs (Yamanaka, 2009). CD8^+^ cytotoxic T cells and NK cells directly target and kill tumor cells (Kim and Cantor, 2014). However, the TIP analysis showed that patients in cluster-2 had a decreased immune activity score for the infiltration of immune cells but an increased score in the steps of cancer cell recognition and killing. This result corresponds to the better outcomes for cluster-2 patients. Glioma is characterized by a “clod” tumor, and immune cells are trapped in the stroma rather than penetrating the tumor parenchyma (Chen and Mellman, 2017; Jackson et al., 2019). Combined with the TIP findings, we found that the effectiveness of anti-tumor immune response may depend on the “effective immune cells”. Therefore, methods to increase “effective immune cells” in LGG will be a new direction of immunotherapy.

Based on the DE-CRLs between different cuproptosis subgroups, we then constructed a 3 CRL prognostic signature, including CRNDE, HAR1A, and FAM181A-AS1, to predict the individualized treatment through the LASSO regression. CRNDE is involved in cancer progression, neuronal differentiation, gametogenesis, and other developmental processes (Han et al., 2017). CRNDE also plays a regulatory role in temozolomide chemoresistance to glioma (Zhao et al., 2021). HAR1A acts as a tumor suppressor in many cancer types. Chen et al. (2020) demonstrated that lower HAR1A expression may result in a worse outcome for glioma patients (Waters et al., 2021). The expression level of lncRNA FAM181A-AS1 correlates with advanced tumor stage and survival of glioma (Jiang and Chen, 2020).

We further demonstrated that patients in the high- and low-risk groups exhibited significantly different survival outcomes. The AUC values of the ROC plots demonstrated the reliability and accuracy of the prognostic signature. The effectiveness and stability of the prognostic signature were validated in the TCGA and CGGA cohorts. Univariate and multivariate Cox regression analyses showed that the risk score was an independent prognostic factor in LGG. Nomogram and calibration plots also showed that the prognostic signature had excellent predictive power.

As an important component of LGG treatment, chemotherapy has attracted more attention. The present research evaluated four chemotherapeutic drug sensitivities in each LGG sample. Temozolomide is a classic chemotherapeutic drug that is used for the treatment of glioma. It is characterized as easy to administer and better tolerated (van den Bent, 2015). Tamoxifen is an estrogen receptor modulator that induces cell death in glioma (Harmalkar et al., 2015). The combined administration of tamoxifen and temozolomide was well tolerated (Carrabba et al., 2013). Bleomycin functions by inducing single- and double-stranded DNA breaks, which is similar to ionizing radiation (Mathews et al., 2012). Research showed that the effect of bleomycin on F98 glioma cells was stronger than temozolomide (Gederaas et al., 2015). One large study revealed that vinblastine showed low toxicity and maintained the quality of life in pediatric LGG (Lassaletta et al., 2016). The predicted IC_50_ values of the above four chemotherapeutic drugs are lower in cluster-1 or high-risk groups, which indicated more sensitivity to the four chemotherapeutic drugs. According to the RTOG 9802 results, LGG patients had increased overall survival after adjuvant chemotherapy and radiation. The chemotherapy drugs procarbazine, lomustine, and vincristine (PCV) were the mainstream treatment strategy for improving LGG patients’ survival rates (Bell et al., 2020). However, we could not assess the sensitivity to PCV in LGG patients due to the limited candidate drugs in the predictive algorithm.

Although ICB therapy has become a promising treatment strategy against a variety of tumors, only a minority of patients obtain favorable benefits from it (Hsu et al., 2021). PD-1, PD-L1, and CTLA-4 are the main immune checkpoint molecules in glioma immunotherapy (Topalian et al., 2015). However, response to anti-PD-1/PD-L1 therapy commonly ranges from 10 to 40% (Zou et al., 2016). Due to the characterization of glioma as a “cold tumor”, the response frequency may be lower (Chen and Mellman, 2017). A clinical trial of recurrent glioma showed that only 8% of patients exhibited dramatic responses to anti-PD-1 therapy (Filley et al., 2017). Therefore, preliminary screening for patients who are sensitive to ICB therapy using a bioinformatics method may provide optimal clinical treatment. The present study found that LGG patients with higher immune cell infiltration, and TMB levels, may produce more clinical responses to ICB therapy. PD-1 and its ligands PD-L1/PD-L2 are the most comprehensively studied immune checkpoint molecules (Qi et al., 2020). CTLA-4 is the first ICP molecule that was used in ICB therapy, its also effect on the CD80 and CD86 expressed by dendritic cells (Ohue and Nishikawa, 2019). Research found that combining select therapies with IDO1, LAG-3, and TIM-3 blockade tend to benefit against tumor growth (Huang et al., 2015; Kim et al., 2017; Zhai et al., 2018). In this study, the expression of these ICP regulators was increased in the high-risk group. The above findings may provide a means for the effective application of ICB therapy for LGG.

Here are some limitations in the current work. First, the sample size was relatively small as a validation group. Second, we selected CRGs from recently published research. With further study of cuproptosis, an increasing number of CRGs may be identified. Finally, this study was based on bioinformatics analysis, and further experimental studies *in vitro* and *in vivo* are needed.

## Conclusion

In summary, we identified two cuproptosis subtypes in LGG with different outcomes, clinicopathological features, and immune landscapes. We also constructed and validated a cuproptosis-related signature that exhibited robust capacity in predicting the survival outcomes of LGG patients. Notably, we also evaluated chemotherapy sensitivity and ICB treatment responsiveness in LGG patients. This study provides a new reference for the chemotherapy and ICB treatment of LGG and may be beneficial in individualized treatment strategies.

## Data Availability

The original contributions presented in the study are included in the article/Supplementary Material, further inquiries can be directed to the corresponding authors.

## References

[B1] Carrabba G. Lanfranchi G. Menghetti C. Rampini P. Caroli M. (2013). Continuous tamoxifen and dose-dense temozolomide in recurrent glioblastoma. Anticancer Res. 33, 3383–3389. PubMed Abstract | Google Scholar 23898108

[B2] Bell E. H. Zhang P. Shaw E. G. Buckner J. C. Barger G. R. Bullard D. E. (2020). Comprehensive genomic analysis in nrg oncology/RTOG 9802: A phase III trial of radiation versus radiation plus procarbazine, lomustine (CCNU), and vincristine in high-risk low-grade glioma. J. Clin. Oncol. 38, 3407–3417. 10.1200/jco.19.02983 PubMed Abstract | 10.1200/jco.19.02983 | Google Scholar 32706640 PMC7527157

[B3] Brat D. J. Verhaak R. G. Aldape K. D. Yung W. K. Salama S. R. Cooper L. A. (2015). Comprehensive, integrative genomic analysis of diffuse lower-grade gliomas. N. Engl. J. Med. 372, 2481–2498. 10.1056/NEJMoa1402121 PubMed Abstract | 10.1056/NEJMoa1402121 | Google Scholar 26061751 PMC4530011

[B4] Buccarelli M. D'Alessandris Q. G. Matarrese P. Mollinari C. Signore M. Cappannini A. (2021). Elesclomol-induced increase of mitochondrial reactive oxygen species impairs glioblastoma stem-like cell survival and tumor growth. J. Exp. Clin. Cancer Res. 40, 228. 10.1186/s13046-021-02031-4 PubMed Abstract | 10.1186/s13046-021-02031-4 | Google Scholar 34253243 PMC8273992

[B5] Chen D. S. Mellman I. (2017). Elements of cancer immunity and the cancer-immune set point. Nature 541, 321–330. 10.1038/nature21349 PubMed Abstract | 10.1038/nature21349 | Google Scholar 28102259

[B6] Chen R. Cohen A. L. Colman H. (2016). Targeted therapeutics in patients with high-grade gliomas: Past, present, and future. Curr. Treat. Options Oncol. 17, 42. 10.1007/s11864-016-0418-0 PubMed Abstract | 10.1007/s11864-016-0418-0 | Google Scholar 27334978

[B7] Chen R. Smith-Cohn M. Cohen A. L. Colman H. (2017). Glioma subclassifications and their clinical significance. Neurotherapeutics 14, 284–297. 10.1007/s13311-017-0519-x PubMed Abstract | 10.1007/s13311-017-0519-x | Google Scholar 28281173 PMC5398991

[B8] Chen Y. Guo Y. Chen H. Ma F. (2020). Long non-coding RNA expression profiling identifies a four-long non-coding RNA prognostic signature for isocitrate dehydrogenase mutant glioma. Front. Neurol. 11, 573264. 10.3389/fneur.2020.573264 PubMed Abstract | 10.3389/fneur.2020.573264 | Google Scholar 33329315 PMC7714930

[B9] Choi Y. S. Bae S. Chang J. H. Kang S. G. Kim S. H. Kim J. (2021). Fully automated hybrid approach to predict the IDH mutation status of gliomas via deep learning and radiomics. Neuro. Oncol. 23, 304–313. 10.1093/neuonc/noaa177 PubMed Abstract | 10.1093/neuonc/noaa177 | Google Scholar 32706862 PMC7906063

[B10] Denoyer D. Clatworthy S. A. S. Cater M. A. (2018). 16. Copper complexes in cancer therapy. Mater. Ions Life Sci., 469–506. 10.1515/9783110470734-022 10.1515/9783110470734-022 | Google Scholar 29394035

[B11] Duffau H. (2018). Diffuse low-grade glioma, oncological outcome and quality of life: A surgical perspective. Curr. Opin. Oncol. 30, 383–389. 10.1097/cco.0000000000000483 PubMed Abstract | 10.1097/cco.0000000000000483 | Google Scholar 30124519

[B12] Filley A. C. Henriquez M. Dey M. (2017). Recurrent glioma clinical trial, CheckMate-143: The game is not over yet. Oncotarget 8, 91779–91794. 10.18632/oncotarget.21586 PubMed Abstract | 10.18632/oncotarget.21586 | Google Scholar 29207684 PMC5710964

[B13] Friedman J. Hastie T. Tibshirani R. (2010). Regularization paths for generalized linear models via coordinate descent. J. Stat. Softw. 33, 1–22. 10.18637/jss.v033.i01 PubMed Abstract | 10.18637/jss.v033.i01 | Google Scholar 20808728 PMC2929880

[B14] Ge E. J. Bush A. I. Casini A. Cobine P. A. Cross J. R. DeNicola G. M. (2022). Connecting copper and cancer: From transition metal signalling to metalloplasia. Nat. Rev. Cancer 22, 102–113. 10.1038/s41568-021-00417-2 PubMed Abstract | 10.1038/s41568-021-00417-2 | Google Scholar 34764459 PMC8810673

[B15] Gederaas O. A. Hauge A. Ellingsen P. G. Berg K. Altin D. Bardal T. (2015). Photochemical internalization of bleomycin and temozolomide--*in vitro* studies on the glioma cell line F98. Photochem. Photobiol. Sci. 14, 1357–1366. 10.1039/c5pp00144g PubMed Abstract | 10.1039/c5pp00144g | Google Scholar 26088711

[B16] Geeleher P. Cox N. Huang R. S. (2014). pRRophetic: an R package for prediction of clinical chemotherapeutic response from tumor gene expression levels. PloS one 9, e107468. 10.1371/journal.pone.0107468 PubMed Abstract | 10.1371/journal.pone.0107468 | Google Scholar 25229481 PMC4167990

[B17] Gibb E. A. Brown C. J. Lam W. L. (2011). The functional role of long non-coding RNA in human carcinomas. Mol. Cancer 10, 38. 10.1186/1476-4598-10-38 PubMed Abstract | 10.1186/1476-4598-10-38 | Google Scholar 21489289 PMC3098824

[B18] Han P. Li J. W. Zhang B. M. Lv J. C. Li Y. M. Gu X. Y. (2017). The lncRNA CRNDE promotes colorectal cancer cell proliferation and chemoresistance via miR-181a-5p-mediated regulation of Wnt/β-catenin signaling. Mol. Cancer 16, 9. 10.1186/s12943-017-0583-1 PubMed Abstract | 10.1186/s12943-017-0583-1 | Google Scholar 28086904 PMC5237133

[B19] Hänzelmann S. Castelo R. Guinney J. (2013). Gsva: Gene set variation analysis for microarray and RNA-seq data. BMC Bioinforma. 14, 7. 10.1186/1471-2105-14-7 10.1186/1471-2105-14-7 | Google Scholar PMC3618321 23323831

[B20] Harmalkar M. Upraity S. Kazi S. Shirsat N. V. (2015). Tamoxifen-Induced cell death of malignant glioma cells is brought about by oxidative-stress-mediated alterations in the expression of BCL2 family members and is enhanced on miR-21 inhibition. J. Mol. Neurosci. 57, 197–202. 10.1007/s12031-015-0602-x PubMed Abstract | 10.1007/s12031-015-0602-x | Google Scholar 26109525

[B21] Heagerty P. J. Lumley T. Pepe M. S. (2000). Time-dependent ROC curves for censored survival data and a diagnostic marker. Biometrics 56, 337–344. 10.1111/j.0006-341x.2000.00337.x PubMed Abstract | 10.1111/j.0006-341x.2000.00337.x | Google Scholar 10877287

[B22] Hoshida Y. Brunet J. P. Tamayo P. Golub T. R. Mesirov J. P. (2007). Subclass mapping: Identifying common subtypes in independent disease data sets. PloS one 2, e1195. 10.1371/journal.pone.0001195 PubMed Abstract | 10.1371/journal.pone.0001195 | Google Scholar 18030330 PMC2065909

[B23] Hsu E. J. Cao X. Moon B. Bae J. Sun Z. Liu Z. (2021). A cytokine receptor-masked IL2 prodrug selectively activates tumor-infiltrating lymphocytes for potent antitumor therapy. Nat. Commun. 12, 2768. 10.1038/s41467-021-22980-w PubMed Abstract | 10.1038/s41467-021-22980-w | Google Scholar 33986267 PMC8119481

[B24] Huang R. Y. Eppolito C. Lele S. Shrikant P. Matsuzaki J. Odunsi K. (2015). LAG3 and PD1 co-inhibitory molecules collaborate to limit CD8+ T cell signaling and dampen antitumor immunity in a murine ovarian cancer model. Oncotarget 6, 27359–27377. 10.18632/oncotarget.4751 PubMed Abstract | 10.18632/oncotarget.4751 | Google Scholar 26318293 PMC4694995

[B25] Hugo W. Zaretsky J. M. Sun L. Song C. Moreno B. H. Hu-Lieskovan S. (2016). Genomic and transcriptomic features of response to anti-PD-1 therapy in metastatic melanoma. Cell. 165, 35–44. 10.1016/j.cell.2016.02.065 PubMed Abstract | 10.1016/j.cell.2016.02.065 | Google Scholar 26997480 PMC4808437

[B26] Hwang I. Pan H. Yao J. Elemento O. Zheng H. Paik J. (2020). CIC is a critical regulator of neuronal differentiation. JCI insight, 135826. 10.1172/jci.insight.135826 PubMed Abstract | 10.1172/jci.insight.135826 | Google Scholar 32229723 PMC7253013

[B27] Iasonos A. Schrag D. Raj G. V. Panageas K. S. (2008). How to build and interpret a nomogram for cancer prognosis. J. Clin. Oncol. 26, 1364–1370. 10.1200/jco.2007.12.9791 PubMed Abstract | 10.1200/jco.2007.12.9791 | Google Scholar 18323559

[B28] Jackson C. M. Choi J. Lim M. (2019). Mechanisms of immunotherapy resistance: Lessons from glioblastoma. Nat. Immunol. 20, 1100–1109. 10.1038/s41590-019-0433-y PubMed Abstract | 10.1038/s41590-019-0433-y | Google Scholar 31358997

[B29] Jia Q. Wang J. He N. He J. Zhu B. (2019). Titin mutation associated with responsiveness to checkpoint blockades in solid tumors. JCI insight, 127901. 10.1172/jci.insight.127901 PubMed Abstract | 10.1172/jci.insight.127901 | Google Scholar 31092729 PMC6542599

[B30] Jia Q. Wu W. Wang Y. Alexander P. B. Sun C. Gong Z. (2018). Local mutational diversity drives intratumoral immune heterogeneity in non-small cell lung cancer. Nat. Commun. 9, 5361. 10.1038/s41467-018-07767-w PubMed Abstract | 10.1038/s41467-018-07767-w | Google Scholar 30560866 PMC6299138

[B31] Jiang X. Chen D. (2020). LncRNA FAM181A-AS1 promotes gliomagenesis by sponging miR-129-5p and upregulating ZRANB2. Aging 12, 20069–20084. 10.18632/aging.103391 PubMed Abstract | 10.18632/aging.103391 | Google Scholar 33080570 PMC7655169

[B32] Jouybari L. Kiani F. Islami F. Sanagoo A. Sayehmiri F. Hosnedlova B. (2020). Copper concentrations in breast cancer: A systematic review and meta-analysis. Curr. Med. Chem. 27, 6373–6383. 10.2174/0929867326666190918120209 PubMed Abstract | 10.2174/0929867326666190918120209 | Google Scholar 31533596

[B33] Kim H. J. Cantor H. (2014). CD4 T-Cell subsets and tumor immunity: The helpful and the not-so-helpful. Cancer Immunol. Res. 2, 91–98. 10.1158/2326-6066.Cir-13-0216 PubMed Abstract | 10.1158/2326-6066.Cir-13-0216 | Google Scholar 24778273

[B34] Kim J. E. Patel M. A. Mangraviti A. Kim E. S. Theodros D. Velarde E. (2017). Combination therapy with anti-PD-1, anti-TIM-3, and focal radiation results in regression of murine gliomas. Clin. Cancer Res. 23, 124–136. 10.1158/1078-0432.Ccr-15-1535 PubMed Abstract | 10.1158/1078-0432.Ccr-15-1535 | Google Scholar 27358487 PMC5735836

[B35] Kucharzewski M. Braziewicz J. Majewska U. Gózdz S. (2003). Selenium, copper, and zinc concentrations in intestinal cancer tissue and in colon and rectum polyps. Biol. Trace Elem. Res. 92, 1–10. 10.1385/bter:92:1:1 PubMed Abstract | 10.1385/bter:92:1:1 | Google Scholar 12721399

[B36] Kumthekar P. Raizer J. Singh S. (2015). Low-grade glioma. Cancer Treat. Res. 163, 75–87. 10.1007/978-3-319-12048-5_5 PubMed Abstract | 10.1007/978-3-319-12048-5_5 | Google Scholar 25468226

[B37] Lassaletta A. Scheinemann K. Zelcer S. M. Hukin J. Wilson B. A. Jabado N. (2016). Phase II weekly vinblastine for chemotherapy-naïve children with progressive low-grade glioma: A Canadian pediatric brain tumor consortium study. J. Clin. Oncol. 34, 3537–3543. 10.1200/jco.2016.68.1585 PubMed Abstract | 10.1200/jco.2016.68.1585 | Google Scholar 27573663

[B38] Li S. Gao P. Dai X. Ye L. Wang Z. Cheng H. (2022). New prognostic biomarker CMTM3 in low grade glioma and its immune infiltration. Ann. Transl. Med. 10, 206. 10.21037/atm-22-526 PubMed Abstract | 10.21037/atm-22-526 | Google Scholar 35280380 PMC8908177

[B39] Li T. Fan J. Wang B. Traugh N. Chen Q. Liu J. S. (2017). Timer: A web server for comprehensive analysis of tumor-infiltrating immune cells. Cancer Res. 77, e108–e10. 10.1158/0008-5472.Can-17-0307 PubMed Abstract | 10.1158/0008-5472.Can-17-0307 | Google Scholar 29092952 PMC6042652

[B40] Liberzon A. Subramanian A. Pinchback R. Thorvaldsdóttir H. Tamayo P. Mesirov J. P. (2011)., 27. Oxford, England, 1739–1740. 10.1093/bioinformatics/btr260 Molecular signatures database (MSigDB) 3.0 Bioinformatics PubMed Abstract | 10.1093/bioinformatics/btr260 | Google Scholar 21546393 PMC3106198

[B41] Lu L. Hu Y. Wang C. Jiang F. Wu C. (2021). Methylation and expression of the exercise-related TLR1 gene is associated with low grade glioma prognosis and outcome. Front. Mol. Biosci. 8, 747933. 10.3389/fmolb.2021.747933 PubMed Abstract | 10.3389/fmolb.2021.747933 | Google Scholar 34869584 PMC8635206

[B42] Mariathasan S. Turley S. J. Nickles D. Castiglioni A. Yuen K. Wang Y. (2018). TGFβ attenuates tumour response to PD-L1 blockade by contributing to exclusion of T cells. Nature 554, 544–548. 10.1038/nature25501 PubMed Abstract | 10.1038/nature25501 | Google Scholar 29443960 PMC6028240

[B43] Mathews M. S. Blickenstaff J. W. Shih E. C. Zamora G. Vo V. Sun C. H. (2012). Photochemical internalization of bleomycin for glioma treatment. J. Biomed. Opt. 17, 058001. 10.1117/1.Jbo.17.5.058001 PubMed Abstract | 10.1117/1.Jbo.17.5.058001 | Google Scholar 22612148 PMC3381024

[B44] Mayakonda A. Lin D. C. Assenov Y. Plass C. Koeffler H. P. (2018). Maftools: Efficient and comprehensive analysis of somatic variants in cancer. Genome Res. 28, 1747–1756. 10.1101/gr.239244.118 PubMed Abstract | 10.1101/gr.239244.118 | Google Scholar 30341162 PMC6211645

[B45] Ohue Y. Nishikawa H. (2019). Regulatory T (Treg) cells in cancer: Can Treg cells be a new therapeutic target? Cancer Sci. 110, 2080–2089. 10.1111/cas.14069 PubMed Abstract | 10.1111/cas.14069 | Google Scholar 31102428 PMC6609813

[B46] Peng W. X. Koirala P. Mo Y. Y. (2017). LncRNA-mediated regulation of cell signaling in cancer. Oncogene 36, 5661–5667. 10.1038/onc.2017.184 PubMed Abstract | 10.1038/onc.2017.184 | Google Scholar 28604750 PMC6450570

[B47] Peng Z. Liu C. Wu M. (2018). New insights into long noncoding RNAs and their roles in glioma. Mol. Cancer 17, 61. 10.1186/s12943-018-0812-2 PubMed Abstract | 10.1186/s12943-018-0812-2 | Google Scholar 29458374 PMC5817731

[B48] Qi Y. Liu B. Sun Q. Xiong X. Chen Q. (2020). Immune checkpoint targeted therapy in glioma: Status and hopes. Front. Immunol. 11, 578877. 10.3389/fimmu.2020.578877 PubMed Abstract | 10.3389/fimmu.2020.578877 | Google Scholar 33329549 PMC7729019

[B49] Ritchie M. E. Phipson B. Wu D. Hu Y. Law C. W. Shi W. (2015). Limma powers differential expression analyses for RNA-sequencing and microarray studies. Nucleic Acids Res. 43, e47. 10.1093/nar/gkv007 PubMed Abstract | 10.1093/nar/gkv007 | Google Scholar 25605792 PMC4402510

[B50] Rizvi N. A. Hellmann M. D. Snyder A. Kvistborg P. Makarov V. Havel J. J. (2015). Cancer immunology. Mutational landscape determines sensitivity to PD-1 blockade in non-small cell lung cancer. Sci. (New York, N.Y.) 348, 124–128. 10.1126/science.aaa1348 10.1126/science.aaa1348 | Google Scholar PMC4993154 25765070

[B51] Roh W. Chen P. L. Reuben A. Spencer C. N. Prieto P. A. Miller J. P. (2017). Integrated molecular analysis of tumor biopsies on sequential CTLA-4 and PD-1 blockade reveals markers of response and resistance. Sci. Transl. Med., eaah3560. 10.1126/scitranslmed.aah3560 PubMed Abstract | 10.1126/scitranslmed.aah3560 | Google Scholar 28251903 PMC5819607

[B52] Shaw E. G. Wang M. Coons S. W. Brachman D. G. Buckner J. C. Stelzer K. J. (2012). Randomized trial of radiation therapy plus procarbazine, lomustine, and vincristine chemotherapy for supratentorial adult low-grade glioma: Initial results of RTOG 9802. J. Clin. Oncol. 30, 3065–3070. 10.1200/jco.2011.35.8598 PubMed Abstract | 10.1200/jco.2011.35.8598 | Google Scholar 22851558 PMC3732006

[B53] Snyder A. Makarov V. Merghoub T. Yuan J. Zaretsky J. M. Desrichard A. (2014). Genetic basis for clinical response to CTLA-4 blockade in melanoma. N. Engl. J. Med. 371, 2189–2199. 10.1056/NEJMoa1406498 PubMed Abstract | 10.1056/NEJMoa1406498 | Google Scholar 25409260 PMC4315319

[B54] Steinbrueck A. Sedgwick A. C. Brewster J. T. 2nd Yan K. C. Shang Y. Knoll D. M. (2020). Transition metal chelators, pro-chelators, and ionophores as small molecule cancer chemotherapeutic agents. Chem. Soc. Rev. 49, 3726–3747. 10.1039/c9cs00373h PubMed Abstract | 10.1039/c9cs00373h | Google Scholar 32525153

[B55] Tang X. Jiang F. Wang X. Xia Y. Mao Y. Chen Y. (2022). Identification of the ferroptosis-related long non-coding RNAs signature to improve the prognosis prediction in papillary renal cell carcinoma. Front. Surg. 9, 741726. 10.3389/fsurg.2022.741726 PubMed Abstract | 10.3389/fsurg.2022.741726 | Google Scholar 35310430 PMC8930926

[B56] Topalian S. L. Drake C. G. Pardoll D. M. (2015). Immune checkpoint blockade: A common denominator approach to cancer therapy. Cancer Cell. 27, 450–461. 10.1016/j.ccell.2015.03.001 PubMed Abstract | 10.1016/j.ccell.2015.03.001 | Google Scholar 25858804 PMC4400238

[B57] Tsvetkov P. Coy S. Petrova B. Dreishpoon M. Verma A. Abdusamad M. (2022). Copper induces cell death by targeting lipoylated TCA cycle proteins. Sci. (New York, N.Y.) 375, 1254–1261. 10.1126/science.abf0529 10.1126/science.abf0529 | Google Scholar PMC9273333 35298263

[B58] Tsvetkov P. Detappe A. Cai K. Keys H. R. Brune Z. Ying W. (2019). Mitochondrial metabolism promotes adaptation to proteotoxic stress. Nat. Chem. Biol. 15, 681–689. 10.1038/s41589-019-0291-9 PubMed Abstract | 10.1038/s41589-019-0291-9 | Google Scholar 31133756 PMC8183600

[B59] van den Bent M. J. (2015). Chemotherapy for low-grade glioma: When, for whom, which regimen? Curr. Opin. Neurol. 28, 633–938. 10.1097/wco.0000000000000257 PubMed Abstract | 10.1097/wco.0000000000000257 | Google Scholar 26397230

[B60] Viaccoz A. Lekoubou A. Ducray F. (2012). Chemotherapy in low-grade gliomas. Curr. Opin. Oncol. 24, 694–701. 10.1097/CCO.0b013e328357f503 PubMed Abstract | 10.1097/CCO.0b013e328357f503 | Google Scholar 22913972

[B61] Waters E. Pucci P. Hirst M. Chapman S. Wang Y. Crea F. (2021). HAR1: An insight into lncRNA genetic evolution. Epigenomics 13, 1831–1843. 10.2217/epi-2021-0069 PubMed Abstract | 10.2217/epi-2021-0069 | Google Scholar 34676772

[B62] Weller M. Wick W. Aldape K. Brada M. Berger M. Pfister S. M. (2015). Glioma. Nat. Rev. Dis. Prim. 1, 15017. 10.1038/nrdp.2015.17 PubMed Abstract | 10.1038/nrdp.2015.17 | Google Scholar 27188790

[B63] Wilkerson M. D. Hayes D. N. (2010)., 26. Oxford, England, 1572–1573. 10.1093/bioinformatics/btq170 ConsensusClusterPlus: A class discovery tool with confidence assessments and item tracking Bioinformatics PubMed Abstract | 10.1093/bioinformatics/btq170 | Google Scholar 20427518 PMC2881355

[B64] Xu L. Deng C. Pang B. Zhang X. Liu W. Liao G. (2018). Tip: A web server for resolving tumor Immunophenotype profiling. Cancer Res. 78, 6575–6580. 10.1158/0008-5472.Can-18-0689 PubMed Abstract | 10.1158/0008-5472.Can-18-0689 | Google Scholar 30154154

[B65] Yamanaka R. (2009). Dendritic-cell- and peptide-based vaccination strategies for glioma. Neurosurg. Rev. 32, 265–273. 10.1007/s10143-009-0189-1 PubMed Abstract | 10.1007/s10143-009-0189-1 | Google Scholar 19214609

[B66] Yoshihara K. Shahmoradgoli M. Martínez E. Vegesna R. Kim H. Torres-Garcia W. (2013). Inferring tumour purity and stromal and immune cell admixture from expression data. Nat. Commun. 4, 2612. 10.1038/ncomms3612 PubMed Abstract | 10.1038/ncomms3612 | Google Scholar 24113773 PMC3826632

[B67] Zeng D. Ye Z. Shen R. Yu G. Wu J. Xiong Y. (2021). Iobr: Multi-Omics immuno-oncology biological research to decode tumor microenvironment and signatures. Front. Immunol. 12, 687975. 10.3389/fimmu.2021.687975 PubMed Abstract | 10.3389/fimmu.2021.687975 | Google Scholar 34276676 PMC8283787

[B68] Zhai L. Ladomersky E. Lenzen A. Nguyen B. Patel R. Lauing K. L. (2018). Ido1 in cancer: A gemini of immune checkpoints. Cell.. Mol. Immunol. 15, 447–457. 10.1038/cmi.2017.143 PubMed Abstract | 10.1038/cmi.2017.143 | Google Scholar 29375124 PMC6068130

[B69] Zhang Z. Kattan M. W. (2017). Drawing nomograms with R: Applications to categorical outcome and survival data. Ann. Transl. Med. 5, 211. 10.21037/atm.2017.04.01 PubMed Abstract | 10.21037/atm.2017.04.01 | Google Scholar 28603726 PMC5451623

[B70] Zhao Z. Liu M. Long W. Yuan J. Li H. Zhang C. (2021). Knockdown lncRNA CRNDE enhances temozolomide chemosensitivity by regulating autophagy in glioblastoma. Cancer Cell. Int. 21, 456. 10.1186/s12935-021-02153-x PubMed Abstract | 10.1186/s12935-021-02153-x | Google Scholar 34454479 PMC8399846

[B71] Zou W. Wolchok J. D. Chen L. (2016). PD-L1 (B7-H1) and PD-1 pathway blockade for cancer therapy: Mechanisms, response biomarkers, and combinations. Sci. Transl. Med. 8, 328rv4. 10.1126/scitranslmed.aad7118 PubMed Abstract | 10.1126/scitranslmed.aad7118 | Google Scholar PMC4859220 26936508

