# Supplementary figures and images for: A cuproptosis-related lncRNAs signature for prognosis, chemotherapy, and immune checkpoint blockade therapy of low-grade glioma
Source: Front Mol Biosci. 2022 Aug 17;9:966843. doi: 10.3389/fmolb.2022.966843 (PMC9428515; doi:10.3389/fmolb.2022.966843)

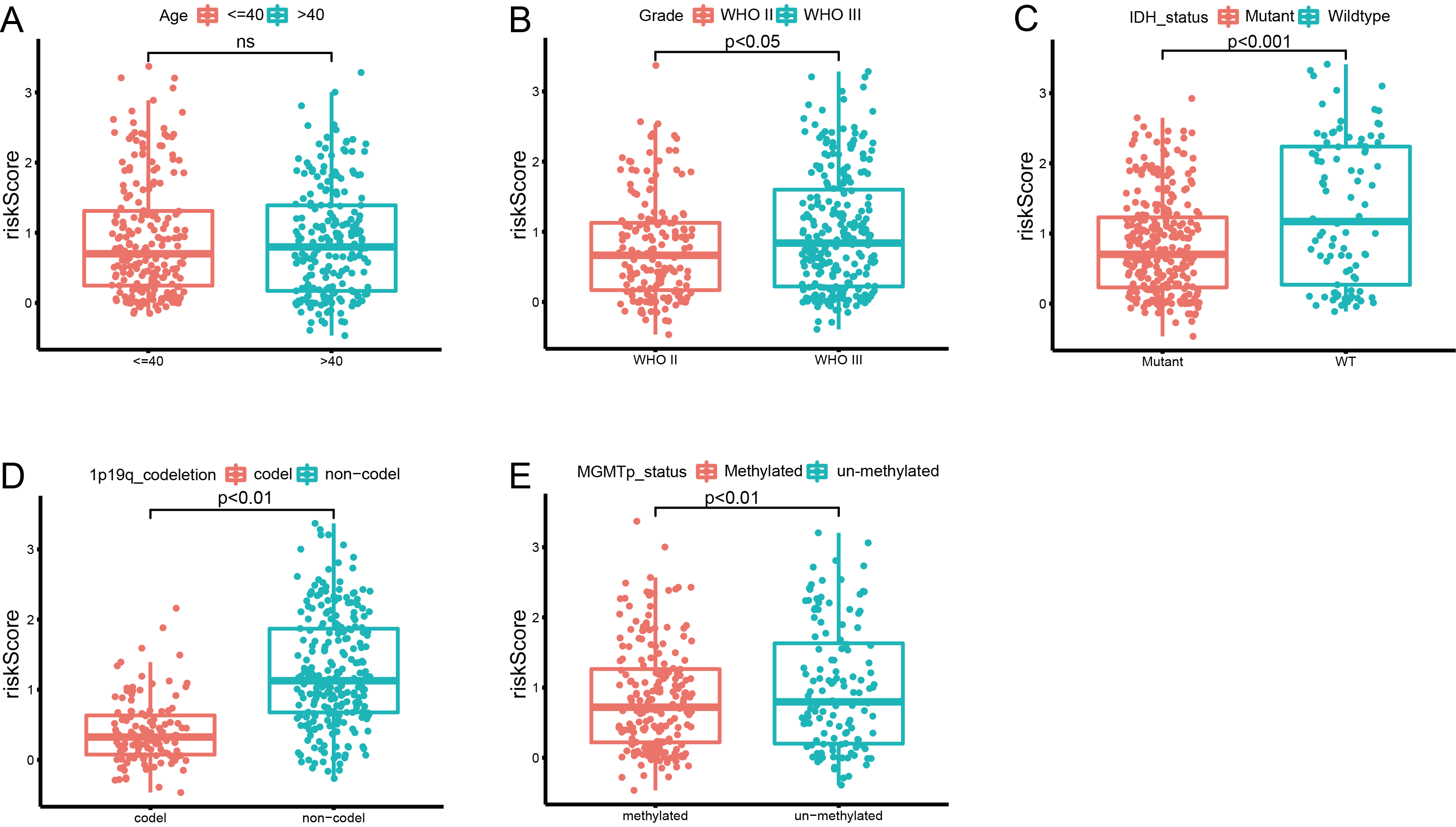

Supplement: Supplementary file 4 [file Image6.TIF]

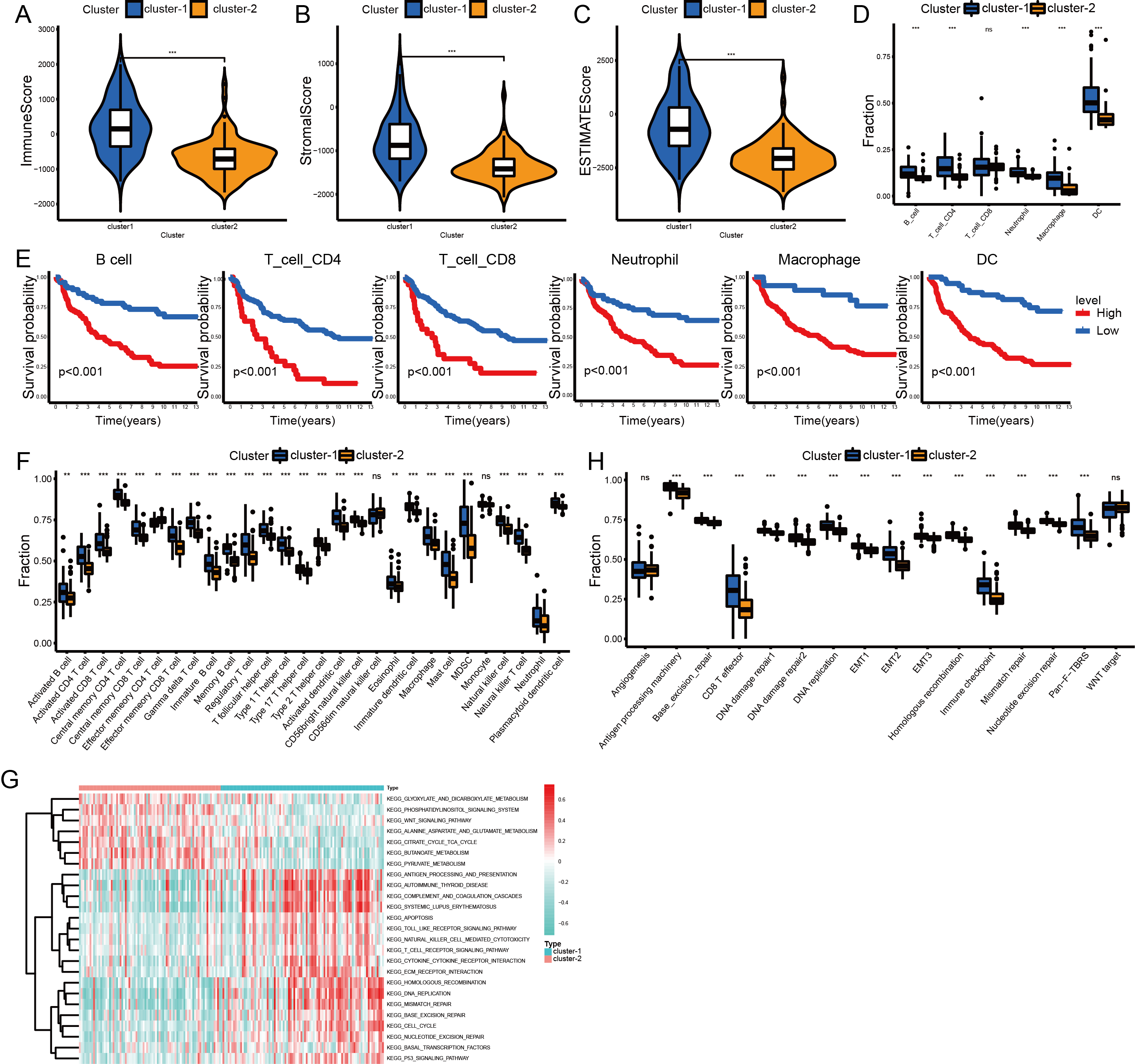

Supplement: Supplementary file 7 [file Image3.TIF]

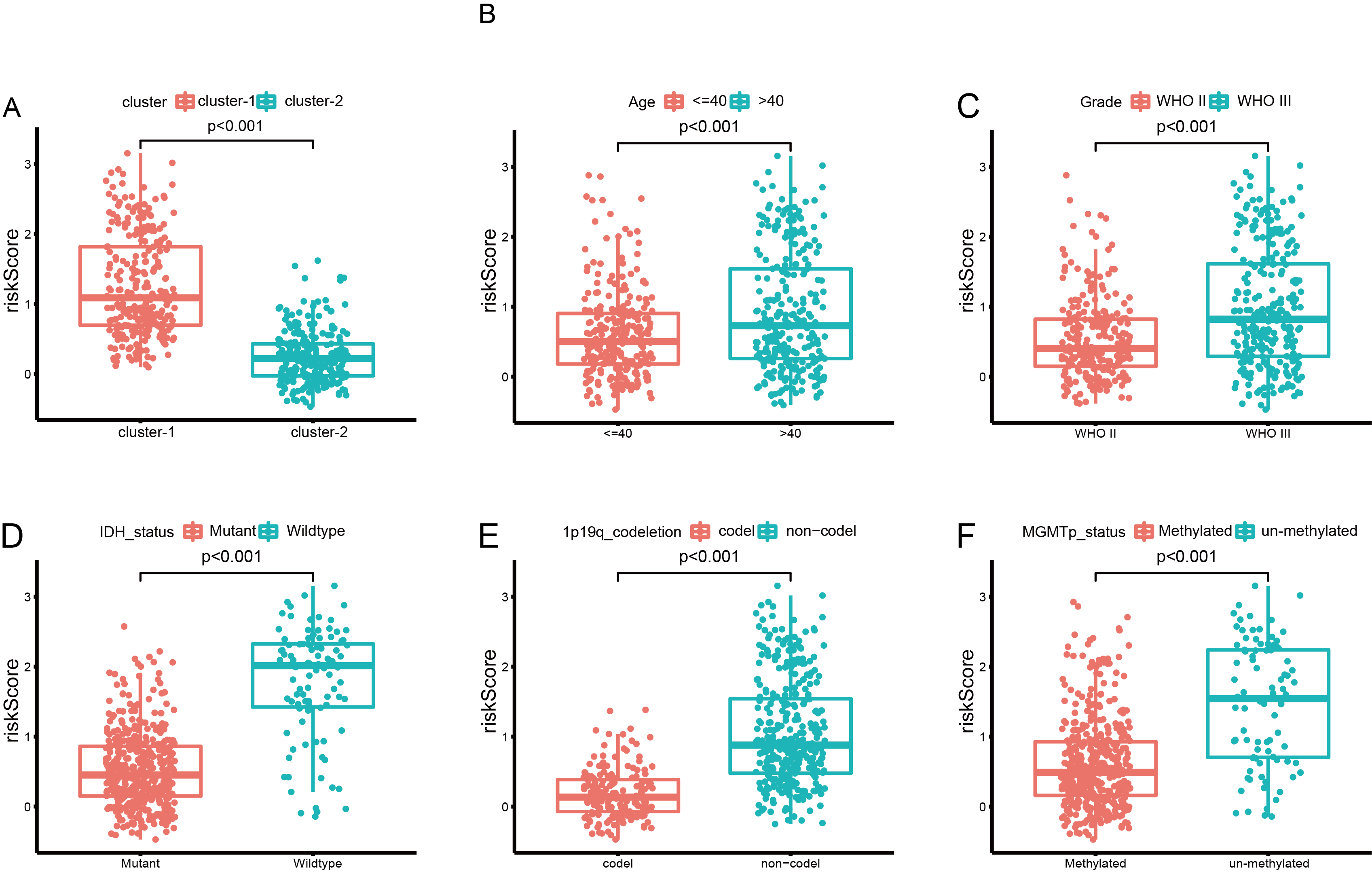

Supplement: Supplementary file 8 [file Image4.TIF]

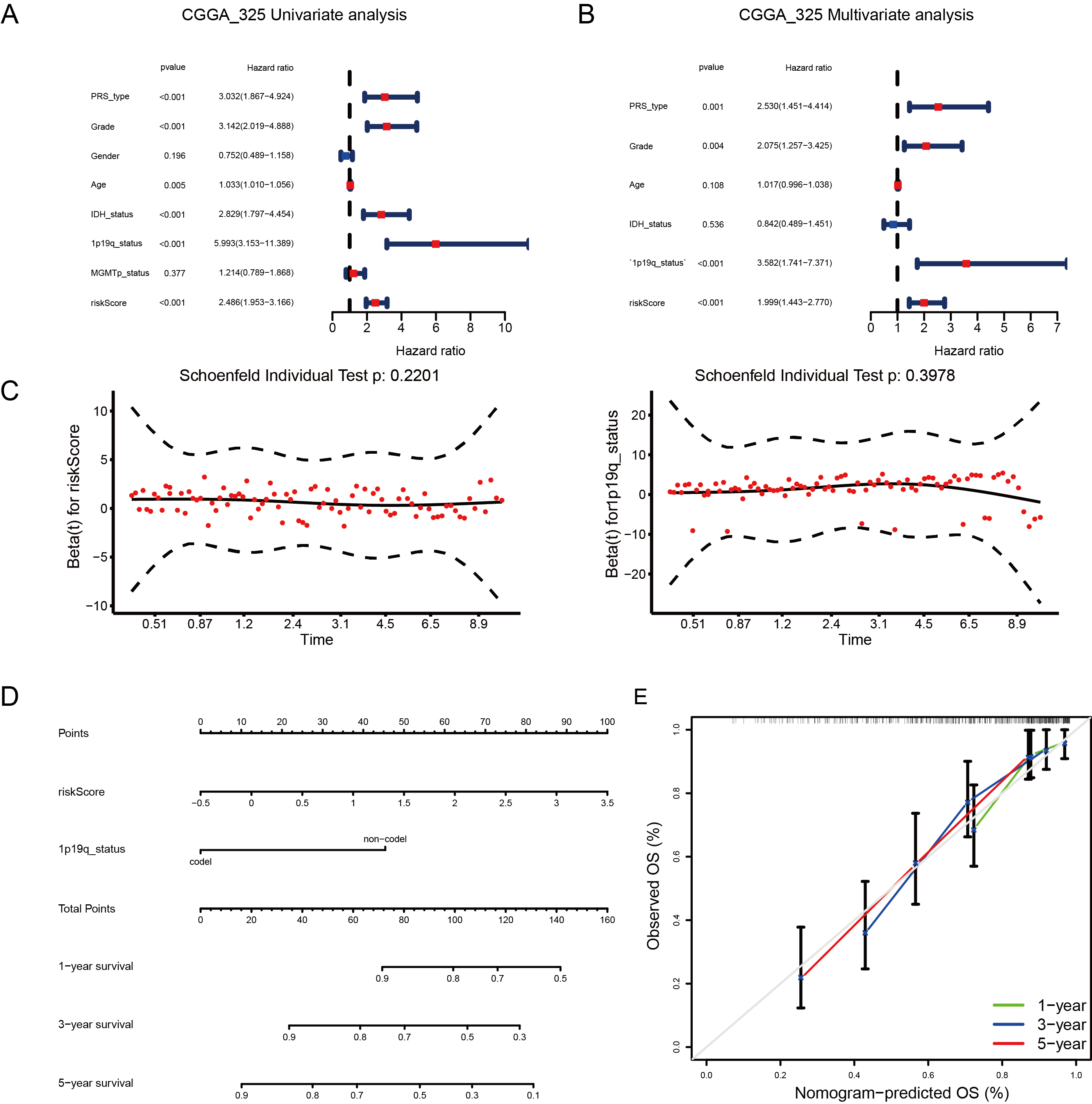

Supplement: Supplementary file 9 [file Image9.TIF]

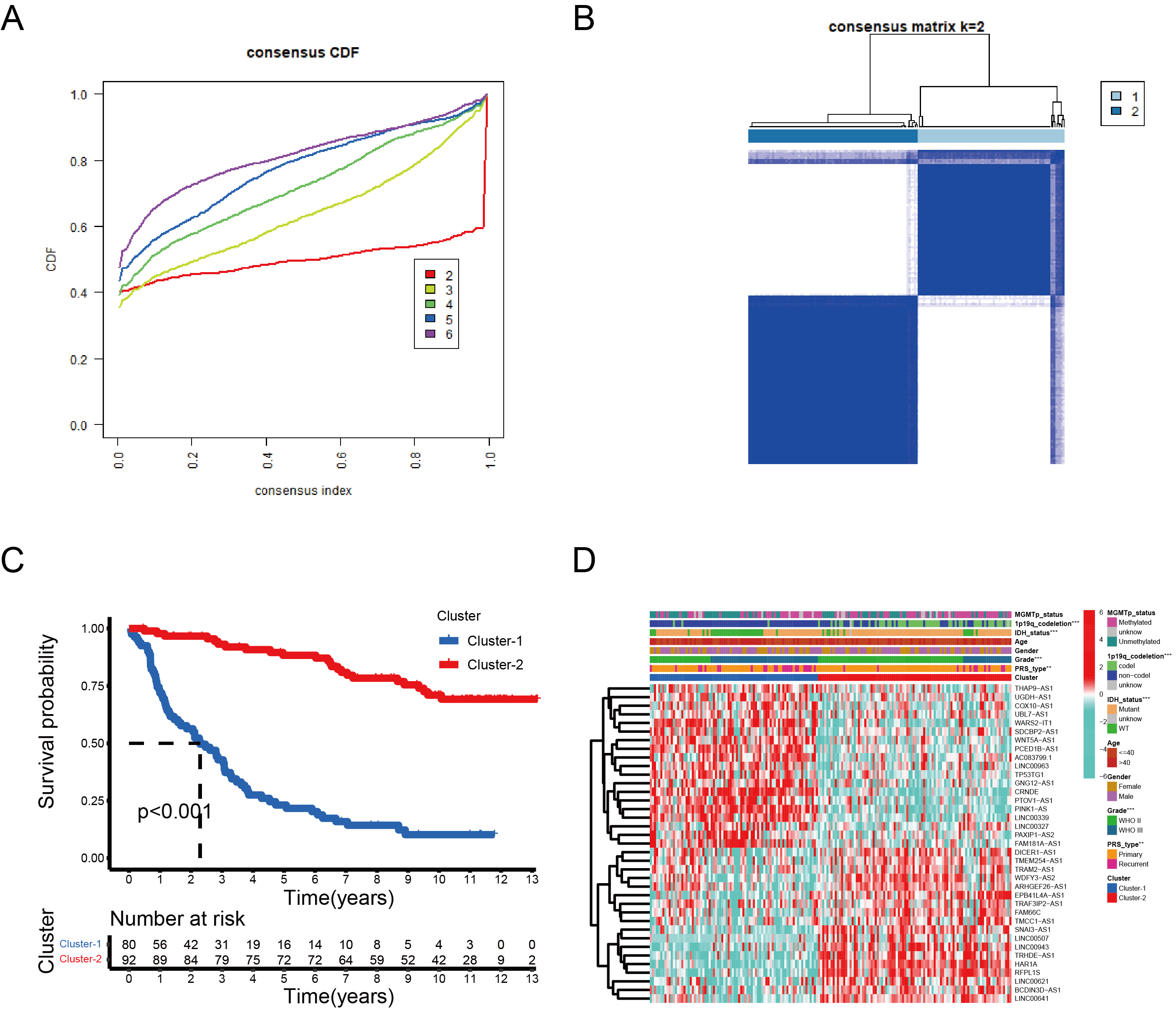

Supplement: Supplementary file 10 [file Image2.TIF]

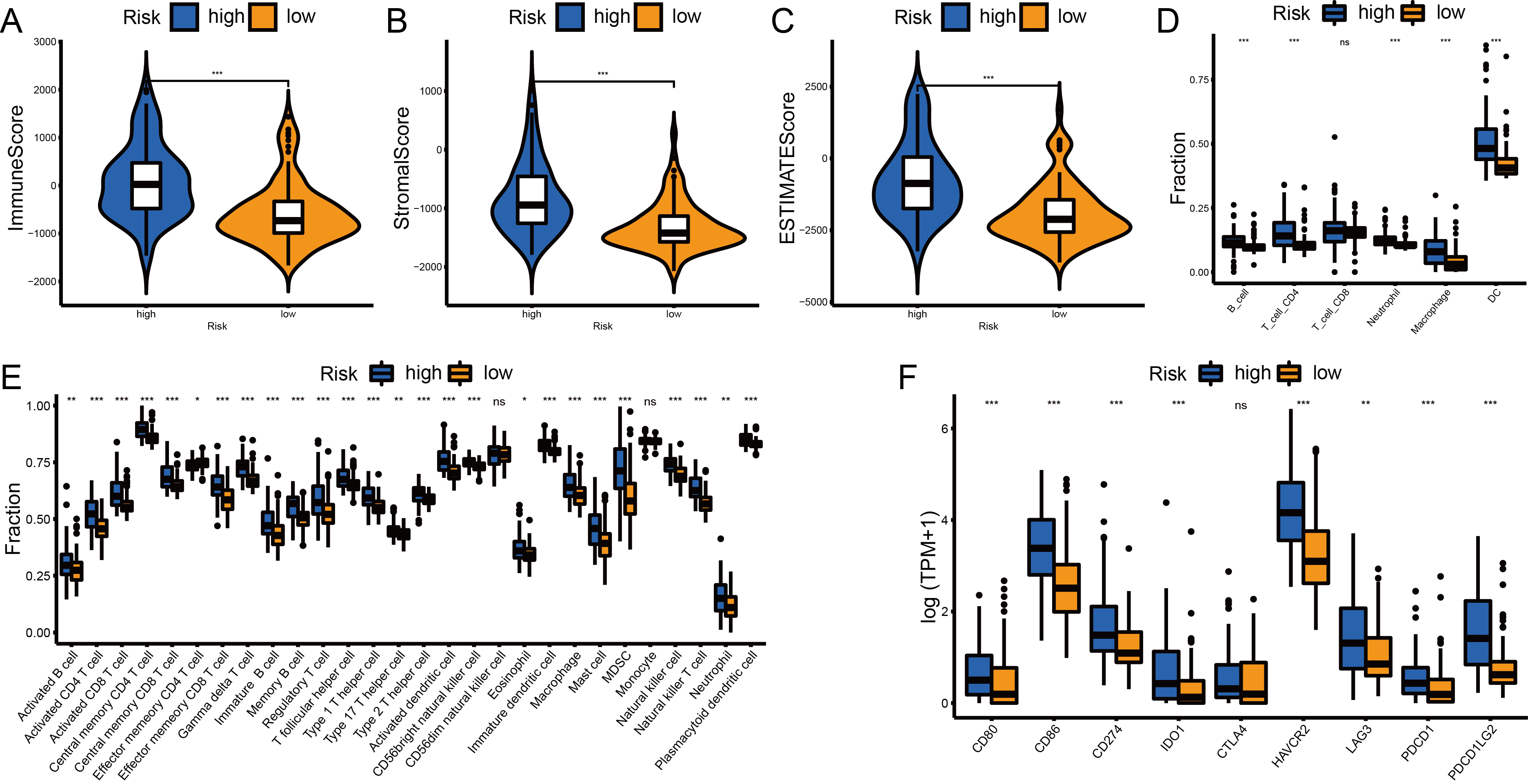

Supplement: Supplementary file 11 [file Image11.TIF]

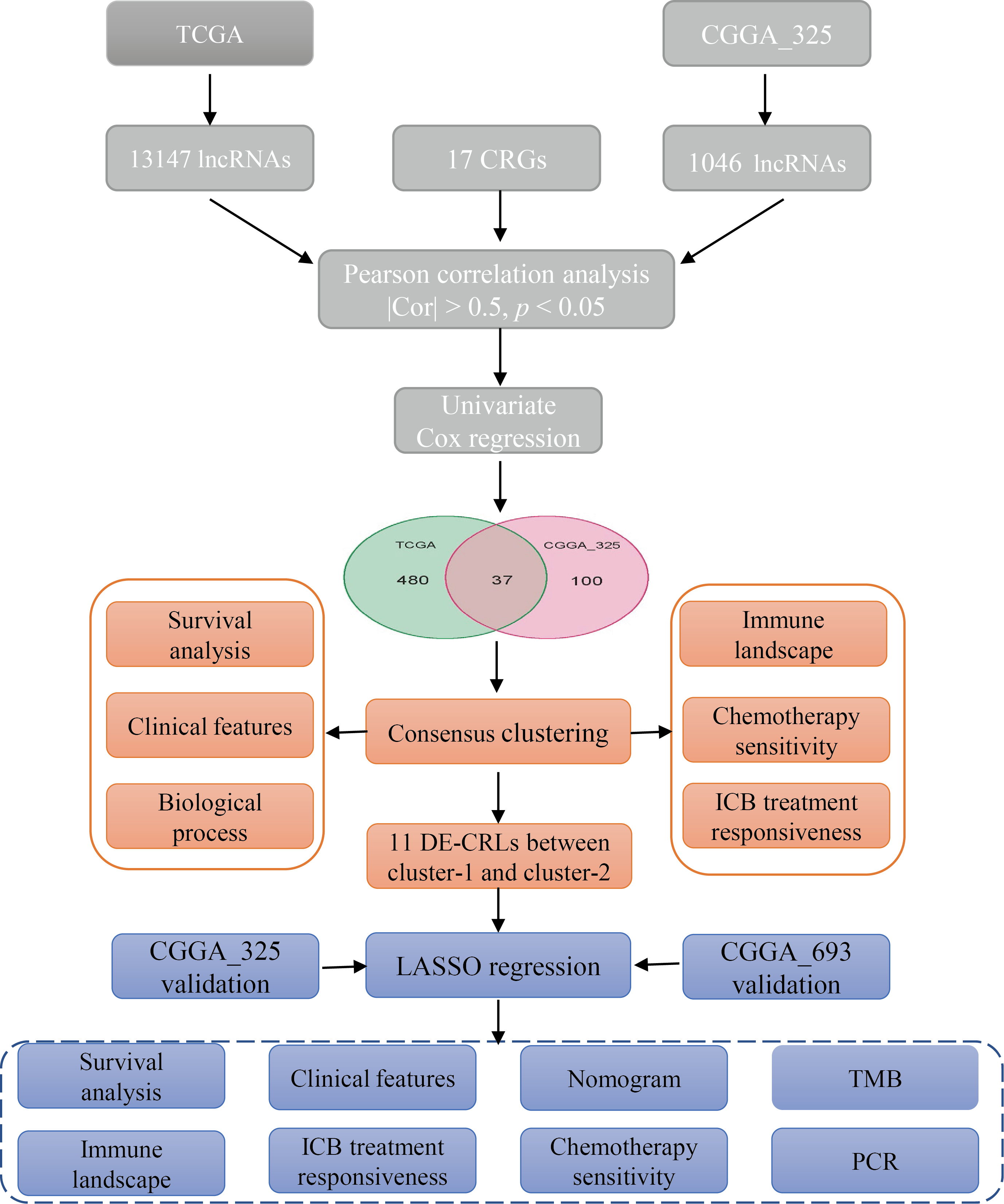

Supplement: Supplementary file 12 [file Image1.TIF]

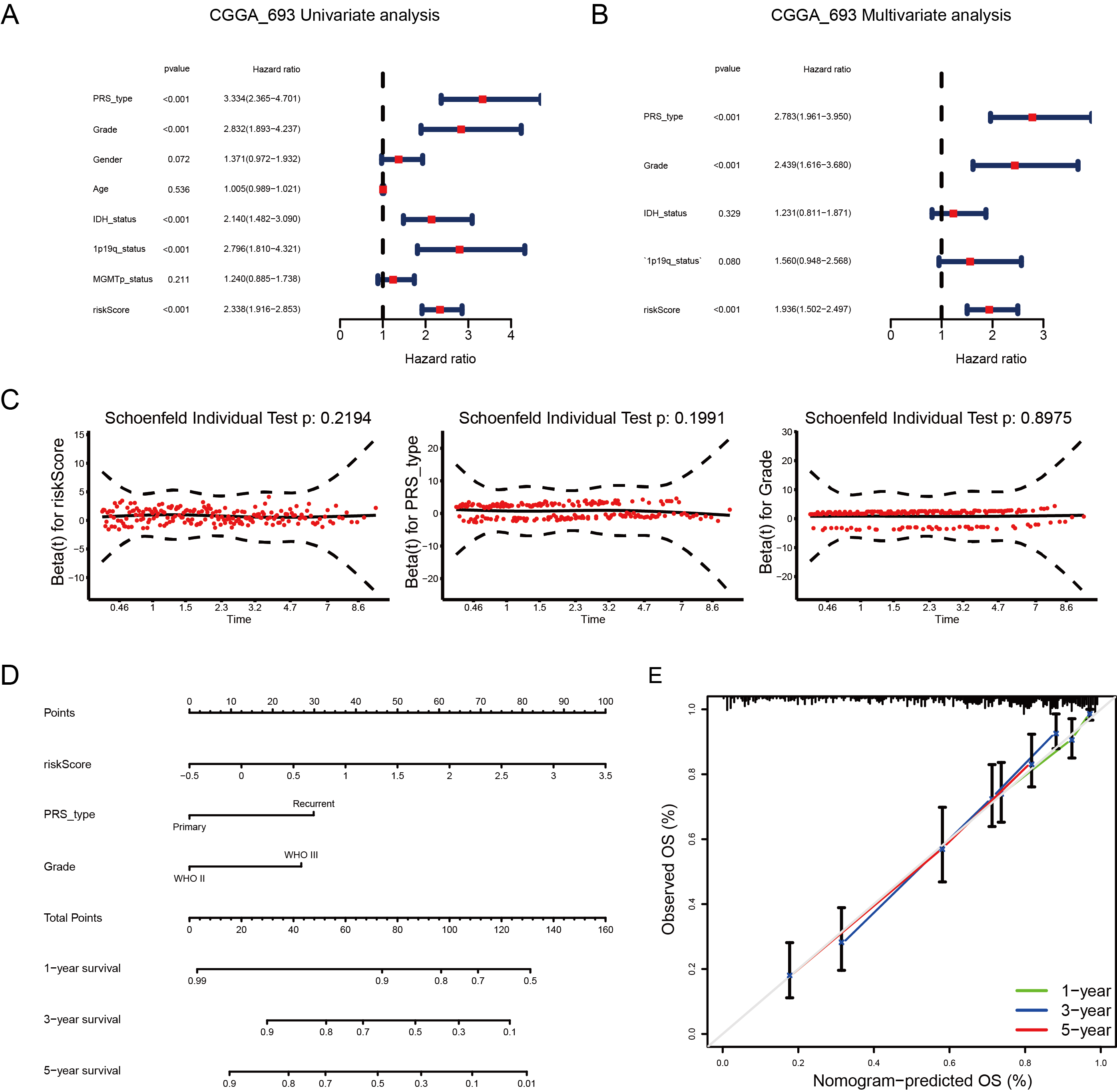

Supplement: Supplementary file 13 [file Image10.TIF]

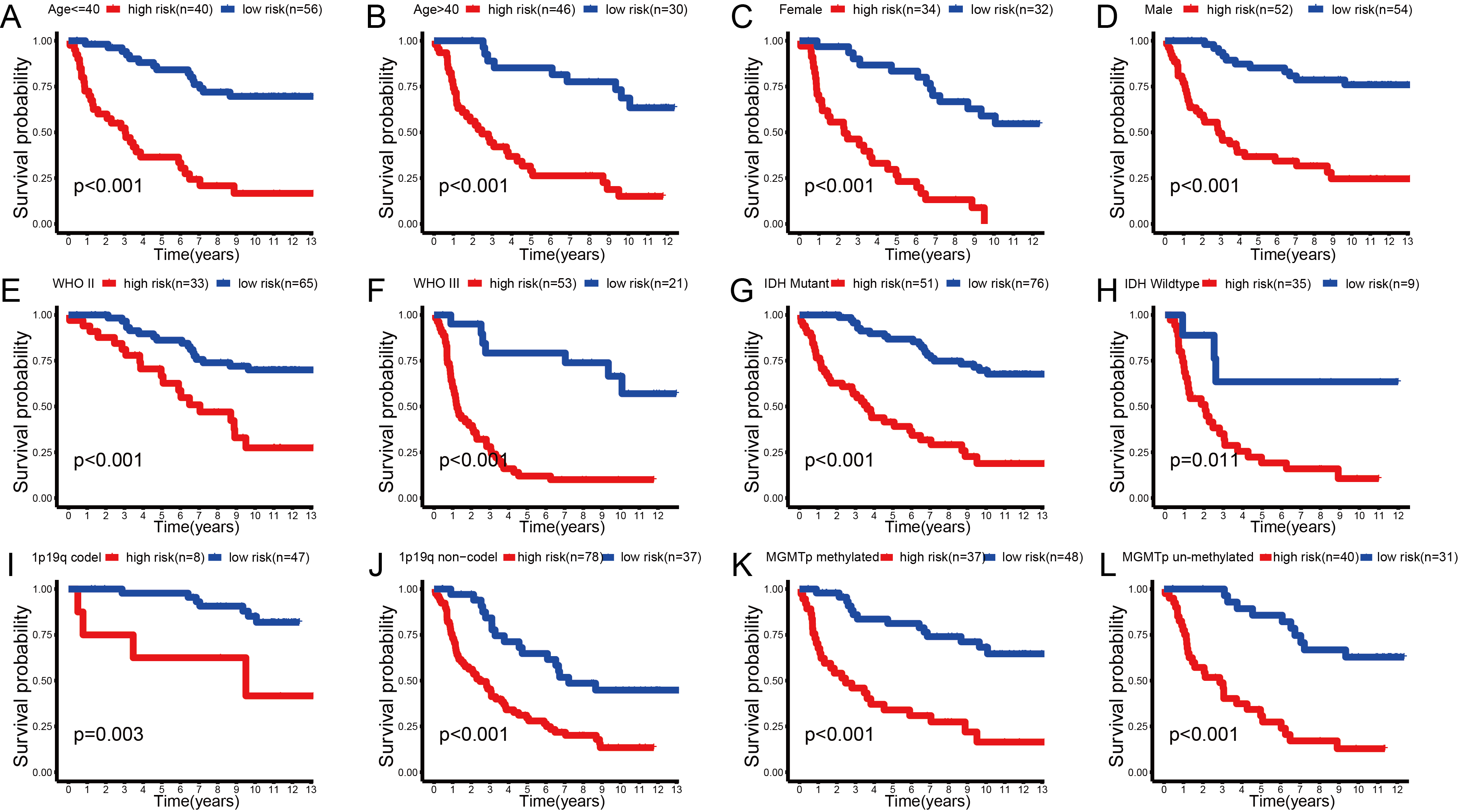

Supplement: Supplementary file 14 [file Image7.TIF]

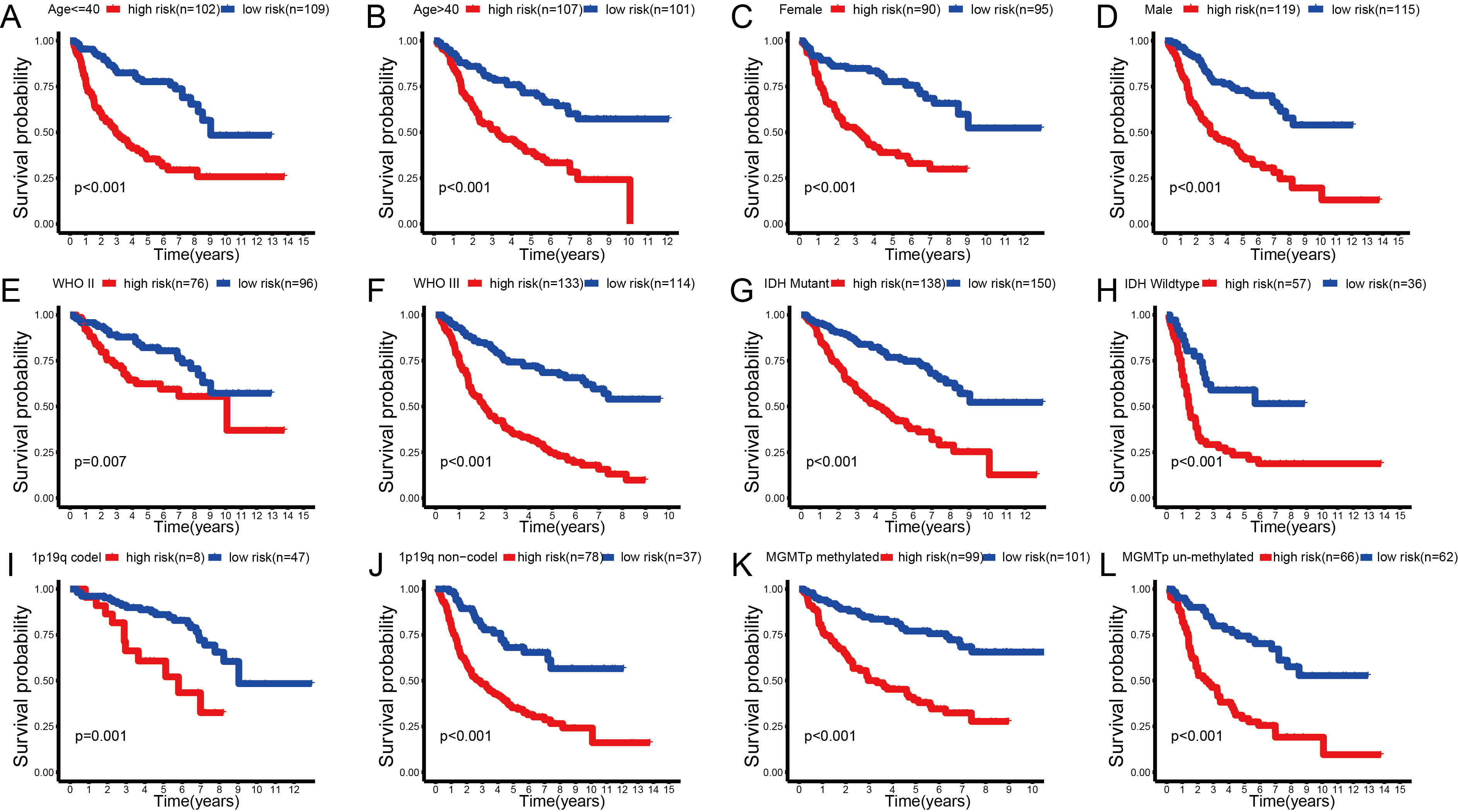

Supplement: Supplementary file 17 [file Image8.TIF]

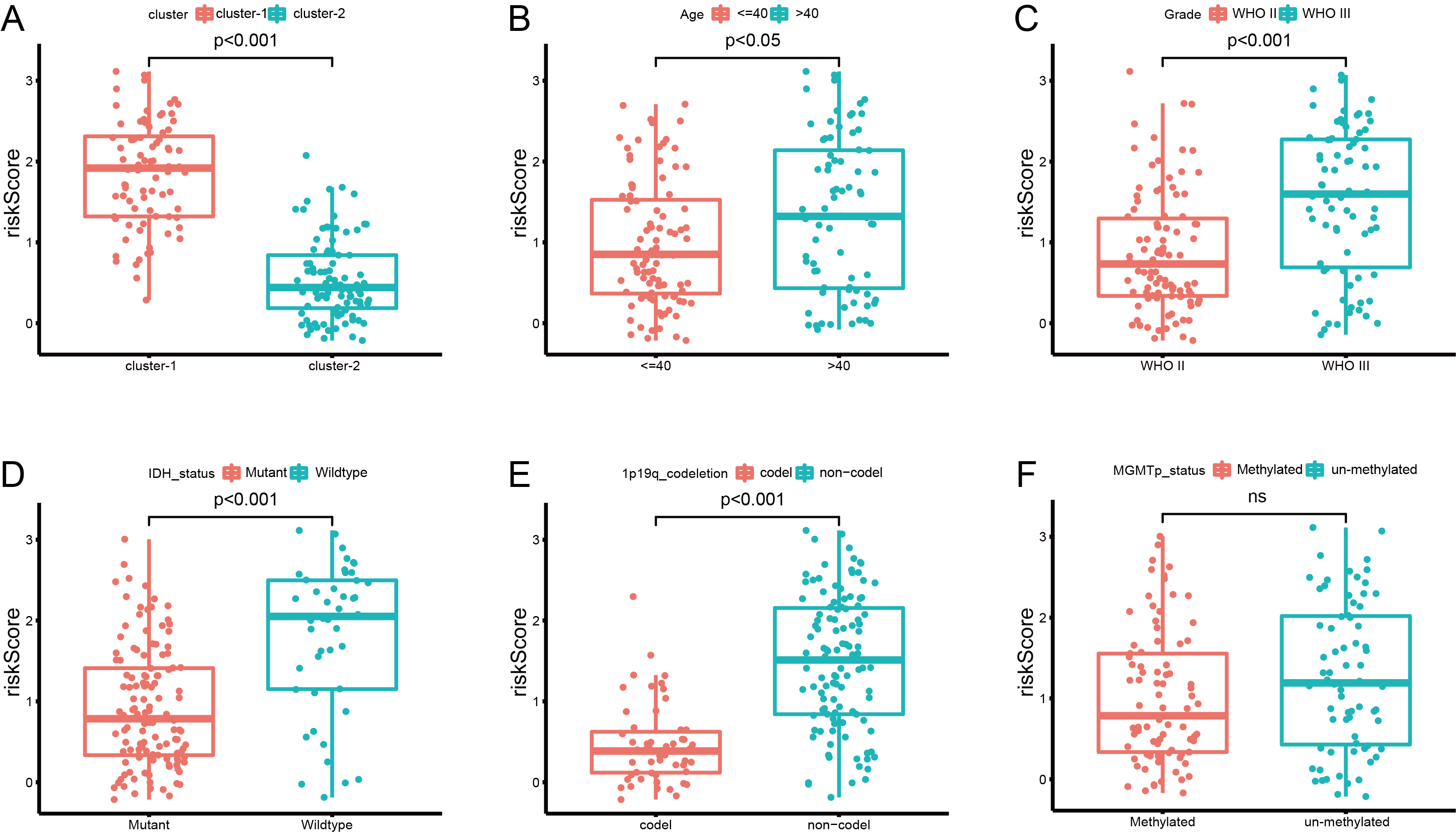

Supplement: Supplementary file 21 [file Image5.TIF]

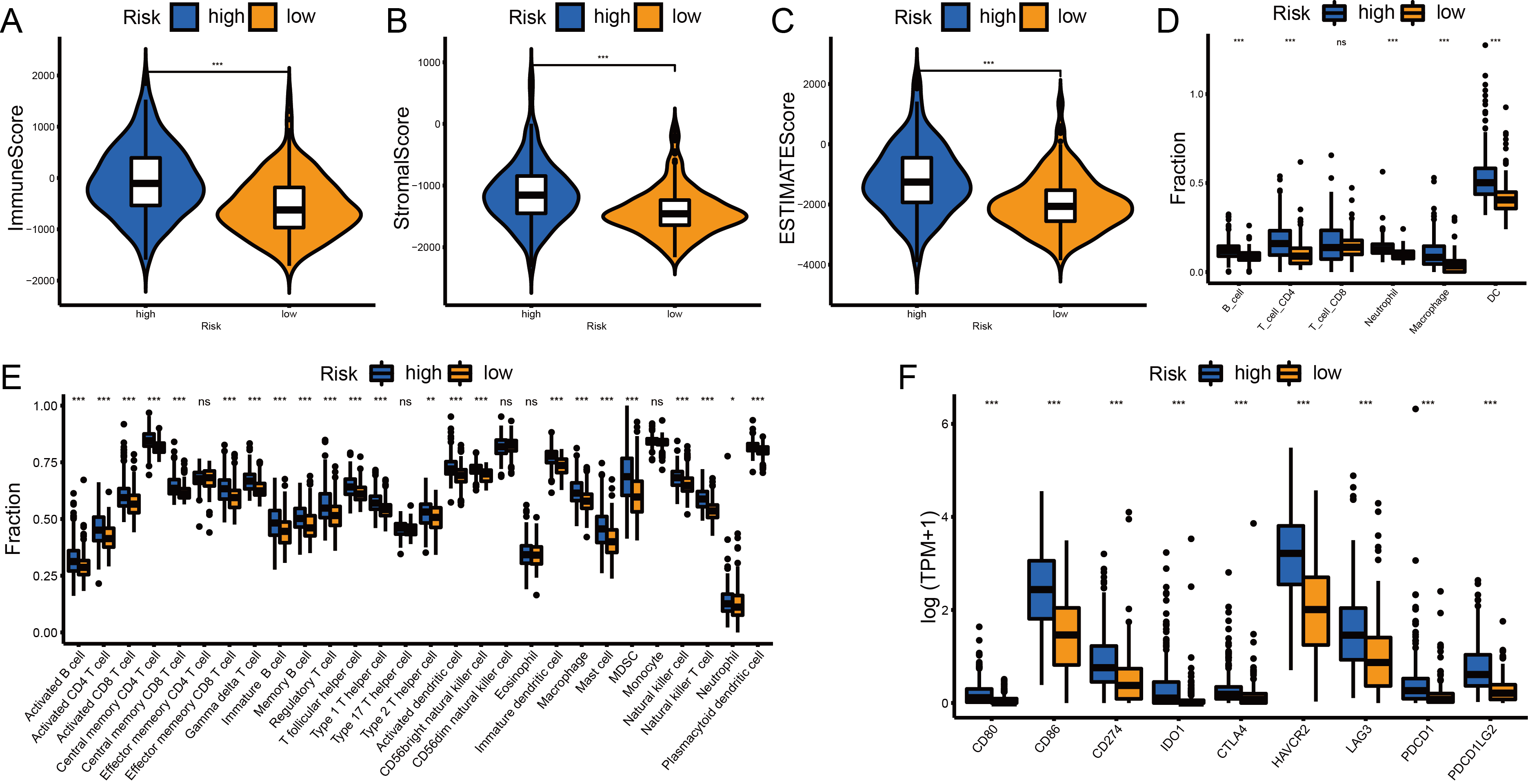

Supplement: Supplementary file 23 [file Image12.TIF]
