# Supplementary material for: A cuproptosis-related lncRNAs signature for prognosis, chemotherapy, and immune checkpoint blockade therapy of low-grade glioma
Source: Front Mol Biosci. 2022 Aug 17;9:966843. doi: 10.3389/fmolb.2022.966843 (PMC9428515; doi:10.3389/fmolb.2022.966843)
Supplement: Supplementary file 5 [file Table1.DOCX]

**Supplementary Table 1 |** Characteristics of patients in TCGA and CGGA datasets.

| **Characteristics** | **TCGA (n=509)** | | | **CGGA (n=172)** | | **CGGA (n=419)** | |
| --- | --- | --- | --- | --- | --- | --- | --- |
| **Age(years)**  <= 40  > 40  **Gender**  Female  Male  **Grade** | | 252  257  227  282 | 96  76  66  106 | | 211  208  185  234 | |  |
| WHO II  WHO III  **IDH status**  Mutant  Wildtype  Unknow  **1p19q codeletion**  codel  non-codel  Unknow  **MGMTp_status** | | 247  262  412  94  3  167  342 | 98  74  127  44  1  55  115  2 | | 172  247  288  93  38  125  256  38 | |  |
| methylated  un-methylated  Unkonw | | 420  89 | 85  71  16 | | 200  128  91 | |  |
