# Supplementary material for: A cuproptosis-related lncRNAs signature for prognosis, chemotherapy, and immune checkpoint blockade therapy of low-grade glioma
Source: Front Mol Biosci. 2022 Aug 17;9:966843. doi: 10.3389/fmolb.2022.966843 (PMC9428515; doi:10.3389/fmolb.2022.966843)
Supplement: Supplementary file 18 [file Table6.DOCX]

**Supplementary Table 6 |** Primer sequences for qRT-PCR.

| **Targets** | **Forward 5′-3′** | **Reverse 5′-3′** |
| --- | --- | --- |
| CRNDE | TAAGGAGGATGCCACTGGAAATG | TAACCTTCTTCTGCGTGACAACT |
| HAR1R | CCAACCCGCAGACCATGTAA | CTGGAACGCCCATCTTCTGT |
| FAM181A-AS1 | GGCTTGGGCTATGACTCCAG | CGGAAAGGGTGCATGGTAGA |
| β-actin | CCTTCCTGGGCATGGAGTC | TGATCTTCATTGTGCTGGGTG |
